# South East Asian Nutrition Surveys II (SEANUTS II) Thailand: triple burden of malnutrition among Thai children aged 6 months to 12 years

**DOI:** 10.1017/S1368980024000053

**Published:** 2024-01-22

**Authors:** Tippawan Pongcharoen, Nipa Rojroongwasinkul, Siriporn Tuntipopipat, Pattanee Winichagoon, Nawarat Vongvimetee, Triwoot Phanyotha, Pornpan Sukboon, Chawanphat Muangnoi, Kemika Praengam, Ilse Khouw

**Affiliations:** 1 Institute of Nutrition, Mahidol University, Nakhon Pathom, Thailand; 2 FrieslandCampina, Amersfoort, The Netherlands

**Keywords:** Nutritional status, Anthropometry, Nutrient intakes, Micronutrient status, Thai children

## Abstract

**Objective:**

This study assessed nutritional status among Thai children using anthropometry, dietary intakes and micronutrient status.

**Design:**

Cross-sectional survey with multi-stage cluster sampling. Body weight and height were measured in all children. Dietary intakes were assessed using 24-h dietary recall. Biochemical assessment was performed in one-third of the children.

**Setting:**

The study was conducted in Thailand’s four geographical regions and Bangkok.

**Participants:**

3478 Thai children aged 0·5–12·9 years.

**Results:**

Stunting showed a downward trend by age group and was most prevalent among infants and toddlers. Overweight and obesity showed a significant upward trend by age group, location and sex and were highest among children aged 7–12·9 years. Risks of inadequate micronutrient intakes (Ca, Fe, Zn, vitamins A, C and D) were high (53·2–93·6 %). Prevalence of Zn and mild vitamin A deficiencies were low; vitamin D and B_12_ deficiencies were nil. Vitamin D insufficiency was significantly higher in the urban area and among girls. Anaemia was very high in infants and toddlers (56·6 and 35·2 %) but showed a significant downward trend by age group. There was an overall high prevalence of Fe deficiency (25 %) *v*. Fe deficiency anaemia (4·2 %) among children aged 4–12·9 years old.

**Conclusions:**

The high prevalence of stunting and anaemia among children aged 0·5–3·9 years and overweight and obesity among children aged 7–12·9 years requires continued attention. While prevalence of biochemical micronutrient deficiencies was not high (except for Fe), high prevalence of dietary inadequacies for several micronutrients warrants further in-depth investigations.

Rapid global industrialisation, especially in Asia, has brought about lifestyle and dietary changes that impact on the nutrition and health of populations^(1)^. For many countries, including Thailand, these changes are rooted in economic, social and political conditions that can lead to a triple burden of malnutrition, that is, the coexistence of overweight/obesity, undernutrition and micronutrient deficiencies. Globally, while the prevalence of stunting in children has declined, the prevalence of overweight and obesity rose rapidly between 2000 and 2019^(2)^. Moreover, the South East Asian region is facing multiple forms of malnutrition due to these dramatic changes^(3)^. Micronutrient deficiencies, especially for vitamin D, Fe, and Zn, are widely prevalent among children^(4)^. Moreover, moderate and severe anaemia among children aged under 5 years as well as pregnant women continues to be a significant public health challenge^(5)^.

Among Thai children aged 0–5 years, the prevalence of stunting and wasting was 10·5 % and 5·4 % in 2016 and 13·3 % and 17·7 % in 2019. On the other hand, the prevalence of overweight and obesity was 8·2 % in 2016 and 9·2 % in 2019^(6,7)^. The coexistence of both undernutrition and overnutrition has also been shown among older children. The prevalence of stunting and wasting in children aged 6–11 years ranged from 3·3 % to 4·1 % in 2009 and from 2·8 % to 4·9 % in 2014. In contrast, the prevalence of overweight and obesity ranged from 6·8 % to 10·4 % in 2009 and 8·8 % to 17·5 % in 2014^(8,9)^.

The South East Asian Nutrition Survey 2010/2011 (SEANUTS I), one of the largest multi-centre nutrition and health studies in South East Asia, was conducted among approximately 17 000 children in Indonesia, Malaysia, Thailand and Vietnam^(10)^. In Thailand, SEANUTS I measured anthropometry, selected micronutrient biomarkers, as well as associated factors, such as dietary intakes, food habits and physical activity. In addition, growth, body composition and cognitive development and performance in country-representative samples of children aged 0·5–12 years were also assessed^(10,11)^. Overall, results demonstrated the existence of double burden malnutrition, with a higher prevalence of stunting and overweight in rural areas and a higher prevalence of obesity among urban children^(11)^. Over half of the children sampled were at risk of inadequate dietary intakes for several micronutrients. Anaemia prevalence was higher among rural children, whereas the prevalence of vitamin D deficiency was more pronounced among urban children. These national level data contributed to initiating the Miracle of 1000 d (First 1000 d) policy and other nutrition-related child health programmes in Thailand^(12)^.

Two nationally representative surveys conducted every 3 and 5 years that have looked into the nutrition situation of Thai children are the Multiple Indicators Cluster Survey (MICS)^(7)^ and the National Health Examination Survey (NHES)^(9)^. However, while these surveys included investigations into child anthropometry, infant and young child feeding practices and eating behaviours, they did not address dietary intakes and entail biochemical assessments.

Consequently, SEANUTS II 2020/2021 aimed at obtaining up-to-date data and deeper insights into the nutritional situation of Thai children beyond that of the Thailand MICS, the NHES and SEANUTS I. Under SEANUTS II, a comprehensive nutritional status assessment was conducted that included anthropometry, body composition, dietary intakes, physical activity and blood biochemistry for assessing micronutrient status. This article presents key descriptive results for anthropometry, dietary intakes and the micronutrient status of Thai children aged 0·5 through 12·9 years from a nationally representative population.

## Methods

### Study design and areas

This cross-sectional study was conducted among Thai children aged 0·5–12·9 years from January to December 2020. It is part of SEANUTS II, a multi-centre study using the same study protocol and carried out in four countries, namely, Indonesia, Malaysia, Thailand and Vietnam^(13)^. Participants were randomly selected using probability proportional to size sampling based on 2017 national population data of the Department of Provincial Administration, Ministry of Interior. A multi-stage cluster sampling approach was used and covered four geographical regions, namely, Central, Northeast, North and South, as well as Bangkok. In the primary stage, systematic sampling of provinces within each region was used to identify three provinces each from the Central and Northeastern regions and two provinces each from the Northern and Southern regions. Bangkok, the capital city, was chosen as one study site due to its different characteristics that may influence lifestyles and is considered an urban area. In the second stage, a random sample of districts nested within the selected provinces was chosen. In the third stage, enumeration areas (EA) within each district by area of residence, that is, municipal (urban) and non-municipal (rural), were selected. In total, eighty-seven EA (thirty-seven EA from municipalities and fifty EA from non-municipalities), covering thirty districts within eleven provinces across Thailand, were selected. Within each EA, a random sample of households was drawn and only one child aged 0·5–12·9 years was recruited from a household.

### Study population

The study population consisted of 3478 children aged 0·5–12·9 years. Apparently healthy children were randomly selected from households within each EA; only one child per household was recruited. Children who were feeling unwell or ill on the day of measurement, those having cognitive and/or physical disabilities diagnosed by physicians, medical histories of serious infection, injuries and/or surgeries requiring hospitalisation during the past 3 months, as well as those having genetic, cardiovascular or respiratory conditions that limit physical activity were all excluded.

The sample size was determined by using the following formula to estimate a population proportion with a specific relative precision^(14)^.

n = Z^2^ P(1 – P) DEFF/(tolerable error)^2^

In this formula, ‘*n*’ is the total number of participants in each group, ‘*Z*’ is the confidence level (*α*: 0·05 and *Z*: 1·96), ‘*P*’ is the prevalence (%) of nutritional status and ‘DEFF’ refers to the estimated design effect (ratio of the actual variance within the sampling method used to that of simple random sampling), estimated at 2·0, and with a tolerable error (level of specificity) of 4 %. The calculation was based on the estimated prevalence of stunting, overweight/obesity, vitamin A deficiency, anaemia and vitamin D insufficiency in each age group from the previous SEANUTS I survey^(11)^. The largest sample size among key indicators was used. The total minimum required sample size was 3186. Taking into account a possible non-response rate of 10 %, the sample size should include up to 3540 children, of which 3478 children aged 0·5–12·9 years were recruited for this study and this represented a 98 % response rate.

### Data collection procedures

Information on the study population and socio-demographic characteristics of the children’s parents or caregivers was obtained by interviewing parents or caregivers using a structured questionnaire. All other assessments including 24-h dietary recall, anthropometric measurements and blood processing were standardised and administered by trained field staff. Blood samples were obtained from a random subsample of one-third of the children, either by finger prick (for children aged 0·5–3·9 years, *n* 524) or venipuncture (for children aged 4–12·9 years, *n* 635). Children who refused to donate a blood sample were replaced.

All data from the questionnaire interview and anthropometric assessment, except for dietary intake, were entered directly into the VIEDOC electronic data capture system. The VIEDOC incorporates a built-in range check and skip condition to avoid error recording.

### Anthropometric measurements

Body weight and height were measured in all children using a standardised anthropometric procedure^(15)^ by well-trained field staff. Technical error of measurement for anthropometry assessment was conducted before and at the midpoint of data collection to ensure intra- and inter-observer variations were minimal among field staff. Measurements were taken in duplicate and average values were used as the final values. In cases where deviation between the two readings was higher than the maximum acceptable difference, a third measurement was taken and the median was used as the final value. Weight of children aged <2 years was measured using a Seca digital weighing scale model 834 (Seca) with a precision of ± 0·1 kg, while weight of children aged ≥2 years was measured using a Seca digital weighing scale model 874 with a precision of ± 0·5 kg. Recumbent length in children aged <2 years was measured in the supine position with a SECA infantometer (SECA 417) to the nearest 0·1 cm. Height was measured in the standing position for children aged ≥2 years using a SECA stadiometer (SECA 217) to the nearest 0·1 cm. The weighing scale was calibrated before data collection and every 3 months thereafter. Anthropometric status was determined using the WHO growth standards for 0–5 years^(16)^ and the WHO growth reference for 5–19 years^(17)^. Z-scores for weight-for-age (WAZ), height-for-age (HAZ), BMI-for-age (BAZ) and weight-for-height (WHZ) were calculated using the software WHO Anthro version 3.2.2 for children aged ≤5 years^(18)^. For children aged >5 years, the WHO AnthroPlus version 1.0.3 software was used^(19)^. Underweight, stunting, wasting and thinness were defined as WAZ < –2 sd, HAZ < –2 sd, WHZ < –2 sd and BAZ < –2 sd, respectively. Overweight and obesity among children aged <5 years old were defined as BAZ > 2 to ≤3 sd and BAZ > 3 sd. Overweight and obesity among children aged 5 years and above were defined as BAZ > 1 to ≤2 sd and BAZ > 2 sd. A total of thirty-four children were excluded from data analysis due to implausible *Z*-score values (WAZ < –5 SD or WAZ > 5 SD, HAZ < –6 SD or HAZ > 6 SD, WHZ < –6 SD or WHZ > 5 SD, or BAZ < –5 SD or BAZ > 5 SD)^(16,17)^.

### Dietary intake assessment

Dietary intake was assessed using 1-d 24-h dietary recall; weekdays and weekend days were included. A parent-proxy report by the mother or the main caregiver through a face-to-face interview was used for children aged 0·5–9·9 years, while a child self-report through a face-to-face interview was used among children aged 10·0–12·9 years. The participants were asked about all foods consumed at home and outside of the home from awakening in the morning to going to bed during the previous day. Portion sizes were estimated using pictorial food models of commonly eaten foods. Estimation of cooked rice (the staple food) was carried out by weighing during the interview. Direct breast-feeding was recorded as breast-feeding occasions, and the amount of breastfed milk was estimated using the method by the Feeding Infants and Toddlers Study 2008^(20)^. The completeness of the 24-h recall form was verified by the field supervisors at each field site. Nutrient intakes were calculated using the INMUCAL-N V 3.1 software^(21)^. The estimated average requirement (EAR) cut-point method was used to derive the prevalence of risk of having inadequate nutrient intakes in a population^(22)^. Except for Ca, EAR values for the other nutrients were taken from the dietary reference intake for Thais, 2020^(23)^. Due to the absence of values in the Thai dietary reference intake, the EAR for Ca was based on harmonised nutrient reference values for populations^(24)^. Nutrient intakes did not include dietary supplements. Prior to data analysis, implausible values of reported energy intake were checked following the predicted total energy intakes (pTEE) method^(25)^. In brief, the pTEE was estimated based on the predicted total energy requirements for moderate physical activity from FAO^(26)^. Thereafter, the SD was calculated from the CV of the reported energy intake among the study population and the CV of the pTEE values from FAO^(26)^. Finally, the CI limits for plausible reported energy intakes were calculated as pTEE ± 2 sd. A total of 155 children with under or over-reporting were excluded.

### Biochemical assessments

For children aged 0·5–3·9 years, a 10 *μ*l blood sample was collected from each child through finger prick and transferred to a microcuvette. Hb concentration was measured by the colorimetric method using the HemoCue (HemoCueHb201+, HemoCue Diagnostics B.V.). For children aged 4 years and above, approximately 10 ml of fasting venous blood per child was collected and aliquoted into the tubes. All blood samples were taken in the morning by phlebotomists. Laboratory supplies for collecting blood samples, including syringes, needles, K_3_EDTA blood collecting tubes, microtubes for sample storage and pipette tips for transferring blood, were randomly sampled from stock to measure Zn levels before starting blood collection. None of them was found to be contaminated. Sample preparation was performed according to the standard protocol by well-trained field staff. Within 1 h of blood collection, serum and plasma were separated using trace-element-free techniques^(27)^. All samples were immediately stored in an ice box at 4–8 °C and then frozen with dry ice within 3–4 h after sampling. Upon finishing fieldwork at each site, serum and plasma aliquots were transported to the laboratory at the Institute of Nutrition and were stored at −80 °C until analysis. Whole blood collected in K_3_EDTA tube was used for determining Hb using the Fluorescent Flow Cytometry. Plasma obtained from the K_3_EDTA tube was used for plasma Zn measurement by inductively coupled plasma MS (ICP-MS). Serum ferritin and vitamin B_12_ concentrations were measured by chemiluminescent microparticle immunoassay (Abbott/Alinity i). C-reactive protein was measured by the immunoturbidimetric method (Abbott/Alinity c). Serum *α*-1-acid glycoprotein and soluble transferrin receptor (sTfR) concentrations were measured by the ELISA. Serum retinol concentration was determined by HPLC and serum 25-hydroxyvitamin D (25(OH)D) by liquid chromatography with tandem MS (LC-MS/MS).

Quality control samples were simultaneously analysed during sample analysis for each parameter. CV of quality control samples were 2·0–2·6 % for Hb, 2·7 % for ferritin, 3·6 % for sTfR, 1·7–2·7 % for C-reactive protein, 8·1 % for *α*-1-acid glycoprotein, 3·5–4·2 % for plasma Zn, 2·9–9·9 % for serum retinol, 4·0–6·7 % for vitamin B_12_ and 2·9–6·8 % and 4·1–5·7 % for 25(OH)D2 and D3, respectively.

Cut-off points for Hb concentrations <110 g/l, <115 g/l and <120 g/l were used to define anaemia in children aged 0·5–<5, 5–11 and 12–12·9 years, respectively^(28)^. Fe deficiency was defined using ferritin corrected for inflammation based on adjustment factors by Thurnham *et al.*^(29)^. Cut-offs for defining Fe deficiency based on serum ferritin (corrected for inflammation) were <12 *μ*g/l for children aged <5 years and <15 *μ*g/l for children aged ≥5 years^(30)^. Fe deficiency erythropoiesis was defined by sTfR concentration >8·3 mg/l^(31)^. Children who had serum ferritin (corrected for inflammation) below cut-offs and/or sTfR > 8·3 mg/l were collectively defined as having Fe deficiency (all stages). C-reactive protein > 5 mg/l and *α*-1-acid glycoprotein > 1 g/l indicated the presence of inflammation^(29)^. Zn deficiency was defined as plasma Zn <70 *μ*g/dl for girls, <74 *μ*g/dl for boys aged ≥10 years and <65 *μ*g/dl for children aged <10 years^(32)^. Vitamin A deficiency was defined as mild for serum retinol between 0·35 and 0·7 *μ*mol/l and as severe for serum retinol <0·35 *μ*mol/l^(33)^. Vitamin B_12_ deficiency was defined as serum vitamin B_12_ < 150 pmol/l (203 pg/ml)^(34)^. Vitamin D insufficiency was defined as serum 25(OH)D between 25 and <50 nmol/l, and vitamin D deficiency where serum 25(OH)D was <25 nmol/l^(35)^.

### Statistical analysis

Statistical analyses were carried out using IBM SPSS Statistics for Windows, version 19.0 (IBM Corp.). All analyses were performed on weighted data by using the ‘Complex Samples’ module. Children were stratified into four age groups, namely, 0·5–0·9 years, 1·0–3·9 years, 4·0–6·9 years and 7·0–12·9 years. Additionally, children aged <5 years were analysed as a separate group. Descriptive analyses were performed and presented as percentage with 95 % CI or means with standard errors. Mean values of anthropometric, biochemical and nutrient intake data were compared between sex and residential areas using ANCOVA with age as a covariate. Test for linear trends was performed using the Mantel–Haenszel test on weighted data to examine whether the prevalence trends in nutritional status increase (or decrease) systematically by the ordered age group. Pearson Chi-square test was used to compare the difference between group percentages for categorical data, such as anthropometry, biochemical status and inadequate nutrient intake by sex and residence. The significance level for all tests was *P*-value less than 0·05 for two-sided tests.

## Results

### Characteristics of participants

A total of 3478 children aged 0·5–12·9 years participated in the study, representing an estimated population of 10 264 568. Among these children, 1101 children were from urban areas, representing an estimated population of 3 215 429, while 2377 children were from rural areas, representing an estimated population of 7 049 141 (Table 1). Selected socio-demographic characteristics are presented in online supplementary material, Supplementary Table 1. Household monthly income and food expenditure in the urban area were significantly higher than those in the rural area (*P* < 0·001). Educational levels among mothers and fathers were also higher in the urban area compared with the rural area (*P* < 0·001).

**Table 1:** Number of children who participated in the study by age group, residence and sex

Age group	0·5–0·9 years		1·0–3·9 years		4·0–6·9 years		7·0–12·9 years		All	
	Sample	Estimated population	Sample	Estimated population	Sample	Estimated population	Sample	Estimated population	Sample	Estimated population
Urban										
Boys	46	72 248	184	262 443	142	397 709	174	924 589	546	1 656 989
Girls	54	100 644	200	240 631	136	315 524	165	901 641	555	1 558 440
All	100	172 892	384	503 074	278	713 233	339	1 826 230	1101	3 215 429
Rural										
Boys	112	175 909	407	580 179	301	817 025	387	2 064 002	1207	3 637 115
Girls	85	158 422	421	498 095	302	761 579	362	1 993 930	1170	3 412 026
All	197	334 331	828	1 078 274	603	1 578 604	749	4 057 932	2377	7 049 141
Urban and rural										
Boys	158	248 157	591	842 622	443	1 214 733	561	2 988 591	1753	5 294 103
Girls	139	259 066	621	738 726	438	1 077 103	527	2 895 571	1725	4 970 465
All	297	507 223	1212	1 581 348	881	2 291 836	1088	5 884 162	3478	10264568

### Nutritional status by anthropometry

**Fig. 1:**
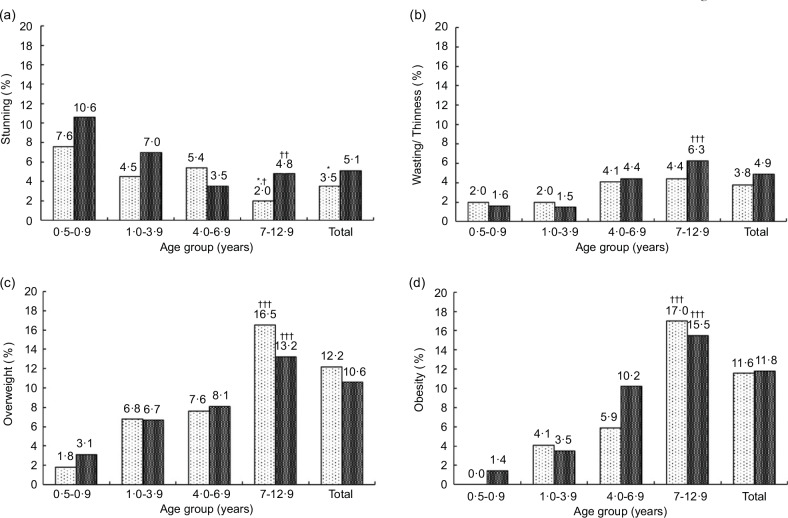
Weighted prevalence of (a) stunting, (b) wasting/thinness, (c) overweight and (d) obesity by age group and residence. *P < 0·05: significant difference between the urban (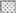) and rural (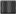) children based on complex samples Pearson Chi-Square. ^†^*P* < 0·05, ^††^*P* < 0·01, ^†††^*P* < 0·001: significance of trend of nutritional status with age group in each urban and rural. Stunting: height-for-age *z*-scores <–2 sd; wasting/thinness: wasting and thinness combined; wasting (<5 years): weight-for-height *z*-scores <–2 sd; thinness (5–12 years): BMI-for-age *z*-scores <–2 sd; overweight: BMI-for-age *z*-scores >2 to ≤3 sd (<5 years) and >1 to ≤2 sd (5–12 years); obesity: BMI-for-age *z*-scores >3 sd (<5 years) and >2 sd (5–12 years)

**Fig. 2:**
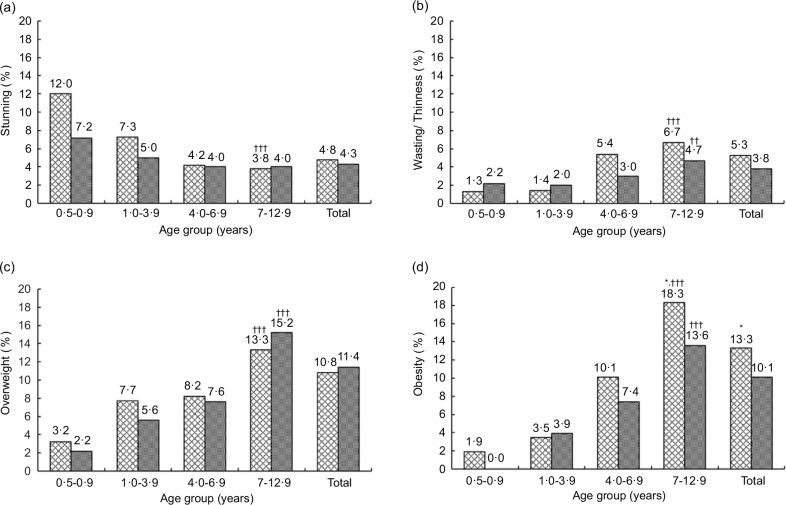
Weighted prevalence of (a) stunting, (b) wasting/thinness, (c) overweight and (d) obesity by age group and sex. *P < 0·05: significant difference between boys (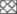) and girls (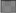) based on complex samples Pearson Chi-Square. ^††^*P* < 0·01, ^†††^*P* < 0·001: significance of trend of nutritional status with age group in each sex. Stunting: height-for-age *z*-scores <–2 sd; wasting/thinness: wasting and thinness combined; wasting (<5 years): weight-for-height *z*-scores <–2 sd; thinness (5–12 years): BMI-for-age *z*-scores <–2 sd; overweight: BMI-for-age *z*-scores >2 to ≤3 sd (<5 years) and >1 to ≤2 sd (5–12 years); (d) obesity: BMI-for-age *z*-scores >3 sd (<5 years) and >2 sd (5–12 years)

Figures 1 and 2 present the prevalence of stunting, wasting, thinness, overweight and obesity by age group and residence, as well as age group and sex, respectively. The prevalence of stunting was highest among rural children aged 0·5–0·9 years (10·6 %). However, a significant difference between urban and rural areas was only observed in the 7–12·9 year age group (*P* < 0·05) (Fig. 1). Boys aged 0·5–0·9 years were worst off in terms of stunting (12·0 % in boys and 7·2 % in girls), but there was no significant difference by sex for the other age groups (Fig. 2). In contrast, the prevalence of stunting was generally low (about 4 %) among children older than 4 years old. For stunting, a significant downward trend by age group existed for both urban and rural areas and among boys (*P*_trend_ <0·001 to <0·05). The prevalence of wasting/thinness was highest among rural children and boys aged 7–12·9 years, but no significant difference by areas of residence or sex. A significant upward trend in wasting/thinness by age group was found in the rural area and among both boys and girls (*P*_trend_ <0·001 to <0·01).

**Fig. 3:**
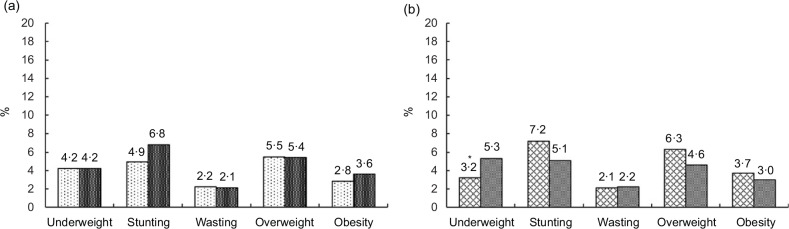
Weighted prevalence of underweight, stunting, wasting, overweight and obesity of children < 5 years old: (a) urban *v*. rural areas, (b) boys *v*. girls. No significant difference between the urban (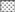) and rural (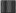) children based on complex samples Pearson Chi-Square. *P < 0·05: significant difference between boys (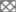) and girls (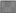) based on complex samples Pearson Chi-Square. Underweight: weight-for-age *z*-scores <–2 sd; stunting: height-for-age z-scores <–2 sd; wasting: weight-for-height *z*-scores <–2 sd; overweight: BMI-for-age *z*-scores >2 to ≤3 sd; obesity: BMI-for-age *z*-scores >3 sd

Alarmingly, the prevalence of both overweight and obesity was above 13 % among children aged 7–12·9 years in both locations and in both sexes, with significantly higher obesity prevalence among boys than girls (*P* < 0·05) (Figs. 1 and 2). There were upward trends by age group for overweight and obesity in both locations and sexes (*P*_trend_ <0·001). There was no significant difference in the prevalence of underweight, wasting, overweight and obesity between the urban and rural areas among children under five years (Fig. 3(a)), whereas a significantly higher prevalence of underweight was found among girls than boys (Fig. 3(b)). Details of anthropometric data by age group, sex and area of residence are given in online supplementary material, Supplementary Tables 2–4.

### Dietary intake

Table 2 shows the percentages of children at risk of having inadequate intakes of protein and key micronutrients. Means of energy intake of children aged 0·5–0·9 years, 1·0–3·9 years, 4·0-6·9 years and 7·0–12·9 years were 732 ± 14, 1213 ± 12, 1512 ± 19 and 1741 ± 18 kcal/d, respectively. Mean intakes of energy, macro–micronutrients and their EAR are presented in online supplementary material, Supplementary Tables 5 and 6. The risk of having inadequate protein intake was very low (Table 2). There was no significant difference in macronutrient intakes and distributions of energy from macronutrients between urban and rural areas for most age groups, except for percentage fat from total energy in the age group of 4–6·9 years and percentage protein from total energy in the oldest age groups (see online supplementary material, Supplementary Table 5). For all age groups, distributions of energy contributions from macronutrients were within the acceptable macronutrient distribution range for Thai children. Despite these observations, it is noticeable that the mean protein intakes for all age groups were 2–3 times higher than EAR. In contrast, mean dietary fibre intakes were only about half of the recommended daily intakes (age in year plus 5 g/d) for respective age groups.

**Table 2:** Weighted percentage of children having risk of inadequate nutrient intakes by residence and sex per age group

		Residence				Sex				All	
		Urban		Rural		Boys		Girls			
Variables	Parameter	%	95 % CI	%	95 % CI	%	95 % CI	%	95 % CI	%	95 % CI
0·5–0·9 years	Protein	8·9	4·5, 16·8	11·8	7·8, 17·3	10·7	6·6, 16·7	10·9	6·6, 17·7	10·8	7·6, 15·1
	Ca	NA	NA	NA	NA	NA	NA	NA	NA	NA	NA
	Fe	71·8	61·6, 80·1	63·9	56·8, 70·5	58·7†	50·6, 66·3	74·2	65·9, 81·1	66·5	60·7, 71·8
	Zn	24·3	16·4, 34·4	23·3	17·7, 30·0	16·7†	11·5, 23·5	30·5	23·1, 39·0	23·6	18·9, 29·1
	Vitamin A	0·0	–	1·5	0·5, 4·5	2·0	0·6, 6·1	0·0	–	1·0	0·3, 3·1
	Vitamin B_12_	NA	NA	NA	NA	NA	NA	NA	NA	NA	NA
	Vitamin C	42·0	32·2, 52·5	41·8	34·9, 49·0	36·0	28·7, 44·0	47·7	39·1, 56·3	41·9	36·1, 47·8
	Vitamin D	NA	NA	NA	NA	NA	NA	NA	NA	NA	NA
1·0–3·9 years	Protein	0·9	0·3, 2·4	0·7	0·3, 1·6	0·6	0·2, 1·6	0·9	0·4, 2·1	0·8	0·4, 1·4
	Ca‡	22·8	18·6, 27·6	22·2	19·3, 25·3	21·9	18·6, 25·6	22·9	19·6, 26·7	22·4	20·0, 25·0
	Fe	31·7	27·0, 36·9	37·9	34·4, 41·5	35·5	31·5, 39·6	36·4	32·4, 40·6	35·9	33·1, 38·9
	Zn	47·7*	42·4, 53·1	55·7	52·1, 59·3	53·2	48·9, 57·4	53·1	48·9, 57·3	53·2	50·2, 56·2
	Vitamin A	17·1	13·3, 21·6	17·1	14·5, 20·0	17·8	14·8, 21·4	16·2	13·3, 19·7	17·1	14·9, 19·5
	Vitamin B_12_	12·6	9·5, 16·6	15·1	12·7, 17·8	17·4††	14·5, 20·8	10·7	8·4, 13·5	14·3	12·3, 16·5
	Vitamin C	38·9	33·8, 44·3	42·3	38·7, 46·0	41·2	37·1, 45·5	41·3	37·1, 45·6	41·2	38·3, 44·3
	Vitamin D	85·3	81·2, 88·6	86·4	83·7, 88·7	85·4	82·1, 88·2	86·7	83·6, 89·3	86·0	83·8, 88·0
4·0–6·9 years	Protein	0·9	0·1, 6·4	0·0	–	0·6	0·1, 3·8	0·0	–	0·3	0·0, 2·0
	Ca‡	72·7	66·2, 78·4	74·7	70·5, 78·5	74·0	68·8, 78·7	74·2	69·5, 78·4	74·1	70·6, 77·3
	Fe	34·0	27·6, 41·0	34·6	30·3, 39·1	32·9	27·7, 38·6	36·1	31·3, 41·1	34·4	30·8, 38·2
	Zn	73·8	67·5, 79·3	67·1	62·6, 71·3	66·1	60·7, 71·2	72·5	67·8, 76·8	69·2	65·6, 72·6
	Vitamin A	46·8	39·9, 53·8	54·6	50·0, 59·2	54·6	49·0, 60·2	49·6	44·4, 54·7	52·2	48·4, 56·0
	Vitamin B_12_	17·7	13·0, 23·6	15·3	12·3, 18·8	15·4	11·8, 19·9	16·7	13·2, 20·9	16·0	13·4, 19·0
	Vitamin C	60·2	53·3, 66·7	64·6	60·1, 68·9	63·9	58·3, 69·1	62·6	57·5, 67·4	63·2	59·5, 66·9
	Vitamin D	92·5	88·5, 95·2	93·0	90·1, 95·1	92·4	88·8, 94·9	93·3	90·5, 95·4	92·8	90·6, 94·6
7·0–12·9 years	Protein	1·4	0·6, 3·4	1·8	1·1, 3·1	1·9	1·1, 3·4	1·5	0·7, 3·0	1·7	1·1, 2·7
	Ca‡	89·3	85·5, 92·2	89·9	87·5, 91·8	88·7	85·8, 91·1	90·7	87·9, 92·9	89·7	87·7, 91·4
	Fe	67·6	62·4, 72·5	61·6	58·0, 65·1	59·0††	54·8, 63·0	68·1	63·9, 72·0	63·5	60·5, 66·3
	Zn	81·3	76·7, 85·1	80·9	77·9, 83·6	75·4†††	71·6, 78·8	86·8	83·6, 89·5	81·0	78·5, 83·2
	Vitamin A	72·3*	67·3, 76·9	66·2	62·7, 69·5	66·9	62·9, 70·7	69·3	65·1, 73·1	68·1	65·2, 70·8
	Vitamin B_12_	31·4	26·7, 36·6	31·2	27·9, 34·6	30·5	26·8, 34·5	32·0	28·1, 36·2	31·3	28·5, 34·1
	Vitamin C	72·8	67·7, 77·4	77·6	74·5, 80·5	78·8†	75·2, 82·0	73·4	69·4, 77·1	76·1	73·5, 78·6
	Vitamin D	96·0	93·3, 97·6	95·8	94·0, 97·0	95·3	93·2, 96·8	96·4	94·4, 97·7	95·8	94·5, 96·9
Overall (0·5–12·9 years)	Protein	1·6	0·9, 2·8	1·7	1·2, 2·4	1·8	1·2, 2·7	1·6	1·0, 2·4	1·7	1·3, 2·3
	Ca‡ (1–12·9 years)	74·9	72·0, 77·7	76·0	74·0, 77·8	74·6	72·2, 76·8	76·8	74·6, 78·9	75·7	74·0, 77·2
	Fe	55·1	51·5, 58·6	52·3	49·9, 54·7	49·5†††	46·7, 52·4	57·0	54·2, 59·8	53·2	51·2, 55·2
	Zn	71·7	68·6, 74·7	71·5	69·4, 73·5	67·2†††	64·6, 69·7	76·2	73·8, 78·3	71·6	69·8, 73·2
	Vitamin A	54·8	51·3, 58·3	53·4	51·0, 55·8	53·7	50·9, 56·4	54·1	51·2, 56·9	53·9	51·9, 55·8
	Vitamin B_12_	25·3	22·1, 28·8	25·0	22·8, 27·3	24·9	22·4, 27·6	25·3	22·7, 28·1	25·1	23·3, 27·0
	Vitamin C	63·4*	60·0, 66·7	67·9	65·7, 70·1	67·7	65·2, 70·2	65·2	62·5, 67·8	66·5	64·7, 68·3
	Vitamin D	93·5	91·7, 94·9	93·7	92·5, 94·7	93·0	91·5, 94·3	94·3	92·9, 95·4	93·6	92·6, 94·5

NA, not available.

* *P* < 0·05: significant difference between the urban and rural children based on complex samples Pearson Chi-Square.

† *P* < 0·05,

†† *P* < 0·01,

††† *P* < 0·001: significant difference between the boys and girls based on complex samples Pearson Chi-Square.

‡ Using the cut point method, for all nutrients: estimated average requirements (EAR) from Thai DRI, except for Ca: EAR from EFSA (Allen LH *et al.* (2019)^(24)^).

There were no significant urban–rural differences in mean micronutrient intakes in all age groups, except for Zn in children aged 4–6·9 years. Sex differences were found for macronutrients and some micronutrients (see online supplementary material, Supplementary Table 6). However, due to differences in requirements by sex, these observed differences are not meaningful. Applying the EAR cut-point method, a very high proportion of children were at risk of inadequate intakes of Ca (75·7 %), Fe (53·2 %), Zn (71·6 %), vitamin A (53·9 %), vitamin C (66·5 %) and vitamin D (93·6 %), but only 25·1 % were at risk of inadequate vitamin B_12_ intake (Table 2). The risk of inadequate Fe intakes among the youngest (0·5–0·9 years) and oldest age (7–12·9 years) groups was higher (66·5 % and 63·5 %, respectively) than for the other age groups. Of concern is the seemingly high inadequate intakes (>60 %) of several micronutrients (i.e. Ca, Zn, vitamin A and vitamin D) among the oldest age groups. For vitamin D, the risk of inadequate dietary intakes was extremely high among all age groups as expected (86·0–95·8 %).

### Micronutrient status by biochemical assessments

Table 3 shows the means for various biochemical assessments. Mean Hb among infants was 108·2–108·8 g/l, which is below the anaemia cut-off for this age group. There were no significant differences in most biochemical parameters by areas of residence or sex, except a significant urban–rural difference for ferritin (corrected) (45·5 ± 2·4 *v*. 55·9 ± 4·1 µg/l, *P* < 0·05) in the oldest age group, as well as a sex difference for vitamin D among children aged 4–6·9 years (boys *v*. girls: 87·4 ± 1·7 *v*. 80·4 ± 2·1 *µ*g/l, *P* < 0·01) and children aged 7–12·9 years (boys *v*. girls: 84·0 ± 1·6 *v*. 72·7 ± 1·5 *µ*g/l, *P* < 0·001).

**Table 3:** Weighted biochemical status by age group, residence and sex

Variables	Residence				Sex					
	Urban		Rural		Boys		Girls		All	
	Mean	se	Mean	se	Mean	se	Mean	se	Mean	se
0·5–0·9 years										
Hb (g/l)	108·8	1·8	108·2	1·3	108·3	1·3	108·4	1·6	108·4	1·0
1·0–3·9 years										
Hb (g/l)	112·5	1·1	113·4	0·8	112·7	0·9	113·6	1·0	113·1	0·7
4·0–6·9 years										
Hb (g/l)	124·7	1·3	125·1	0·8	125·4	1·0	124·5	0·9	125·0	0·7
Ferritin (Thurnham-adjusted) (µg/l)	44·3	5·3	38·9	2·1	40·0	3·6	41·1	2·4	40·5	2·2
Soluble transferrin receptor (mg/l)	7·7	0·5	7·4	0·2	7·7	0·4	7·2	0·2	7·5	0·2
Retinol (µmol/l)	1·4	0·0	1·4	0·0	1·4	0·0	1·4	0·0	1·4	0·0
Vitamin D (nmol/l)	82·8	2·6	84·7	1·6	87·4‡‡	1·7	80·4	2·1	84·1	1·4
Vitamin B_12_ (pmol/l)	650·0	33·0	621·7	20·4	630·0	23·3	631·4	26·3	630·7	17·5
Zn (µg/dl)	85·6	1·0	86·2	0·9	87·5‡	1·0	84·3	0·8	86·0	0·7
7·0–12·9 years										
Hb (g/l)	129·0	0·9	130·0	0·6	129·9	0·7	129·4	0·8	129·7	0·5
Ferritin (Thurnham-adjusted) (µg/l)	45·5†	2·4	55·9	4·1	52·7	2·9	52·7	5·1	52·7	2·9
Soluble transferrin receptor (mg/l)	7·0	0·2	7·2	0·2	7·1	0·2	7·2	0·2	7·1	0·1
Retinol (µmol/l)	1·5	0·0	1·5	0·0	1·5	0·0	1·5	0·0	1·5	0·0
Vitamin D (nmol/l)	76·4	2·1	79·3	1·4	84·0‡‡‡	1·6	72·7	1·5	78·4	1·2
Vitamin B_12_ (pmol/l)	496·5	16·8	516·4	13·1	514·5	15·4	505·8	14·0	510·2	10·4
Zn (µg/dl)	84·8	0·8	86·1	0·6	85·2	0·7	86·3	0·7	85·7	0·5

*Corrected for inflammation by C-reactive protein and *α*-1-acid glycoprotein according to Thurnham *et al.* (2015)^(29)^.

† *P* < 0·05: significant difference between the urban and rural children based on complex samples ANCOVA after correcting for age.

‡ *P* < 0·05,

‡‡ *P* < 0·01,

‡‡‡ *P* < 0·001: significant difference between the boys and girls based on complex samples ANCOVA after correcting for age.

The prevalence of anaemia was alarmingly high among infants aged 0·5–0·9 years (56·6 %) and young children aged 1–3·9 years (35·2 %) (Table 4). However, about 60 % of children in any age group were mildly anaemic (Hb 100–109 g/l). The remaining children were moderately anaemic (Hb 70–99 g/l), and none was severely anaemic according to WHO cut-offs^(28)^ (data not shown). For older children, the prevalence of anaemia was much lower (11·5 % for those aged 4–6·9 years and 6·5 % for those aged 7–12·9 years). Significant downward trends in anaemia prevalence by age were observed in both areas of residence and in both sexes (*P*_trend_ <0·001).

**Table 4:** Weighted prevalence of anaemia and micronutrient deficiencies by age group, residence and sex

	Residence				Sex				All	
	Urban		Rural		Boys		Girls			
Variables	%	95 % CI	%	95 % CI	%	95 % CI	%	95 % CI	%	95 % CI
0·5–0·9 years										
Anaemia§	54·4	38·7, 69·2	57·7	46·4, 68·3	58·7	46·1, 70·3	54·5	41·2, 67·3	56·6	47·4, 65·4
1·0–3·9 years										
Anaemia§	39·6	31·1, 48·7	33·2	27·8, 39·1	36·8	30·3, 43·8	33·4	27·1, 40·4	35·2	30·6, 40·1
4·0–6·9 years										
Anaemia§	15·3	8·2, 26·9	9·8	6·1, 15·4	10·0	5·5, 17·4	13·3	8·1, 21·1	11·5	7·9, 16·6
Fe deficiency (by ferritin, Thurnham-adjusted)||	10·9	6·2, 18·3	12·4	7·9, 19·0	13·6	8·1, 21·9	10·1	6·3, 15·8	11·9	8·3, 16·8
Fe deficiency erythropoiesis (by sTfR)¶	25·6	16·5, 37·5	23·7	17·2, 31·6	24·6	17·1, 34·0	23·9	16·6, 33·3	24·3	18·8, 30·7
Fe deficiency, all stages**	28·4	19·2, 39·8	30·4	23·3, 38·7	29·3	21·3, 38·8	30·4	22·4, 39·8	29·8	24·0, 36·4
Fe deficiency anaemia										
Anaemia with low ferritin, Thurnham-adjusted*†	0·0	–	2·3	1·0, 5·2	1·0	0·3, 4·1	2·3	0·8, 6·0	1·6	0·7, 3·6
Anaemia with ferritin, Thurnham-adjusted and/or sTfR, no adjustment*‡	3·5	1·3, 9·3	5·2	2·8, 9·4	3·2	1·5, 6·7	6·3	3·1, 12·4	4·7	2·8, 7·8
Vitamin A deficiency (mild)	0·0	–	2·1	0·5, 8·0	0·0	–	3·0	0·8, 11·3	1·4	0·4, 5·5
Vitamin A deficiency (severe)	0·0	–	0·0	–	0·0	–	0·0	–	0·0	–
Vitamin B_12_ deficiency	0·0	–	0·0	–	0·0	–	0·0	–	0·0	–
Vitamin D insufficiency	5·5*	2·0, 14·0	1·2	0·4, 3·6	1·8	0·7, 4·8	3·3	1·1, 9·5	2·5	1·2, 5·4
Vitamin D deficiency	0·0	–	0·0	–	0·0	–	0·0	–	0·0	–
Zn deficiency	0·9	0·1, 6·2	0·7	0·2, 2·9	0·9	0·2, 3·8	0·6	0·1, 4·2	0·8	0·3, 2·4
7·0–12·9 years										
Anaemia§	6·3‡‡‡	3·0, 12·7	6·5‡‡‡	4·0, 10·4	6·0‡‡‡	3·4, 10·6	6·9‡‡‡	3·9, 11·8	6·5	4·3, 9·6
Fe deficiency (by ferritin, Thurnham-adjusted)||	3·5‡‡	1·3, 9·0	7·1‡	4·4, 11·2	5·7‡‡	3·1, 10·3	6·3‡	3·5, 11·0	6·0	3·9, 9·0
Fe deficiency erythropoiesis (by sTfR)¶	18·9	12·6, 27·3	21·3	16·6, 26·9	22·5	16·9, 29·2	18·6	13·5, 25·0	20·5	16·6, 25·1
Fe deficiency, all stages**	21·4	14·8, 30·1	23·9	18·9, 29·7	25·3	19·4, 32·3	20·9‡	15·5, 27·5	23·1	19·0, 27·8
Fe deficiency anaemia										
Anaemia with low ferritin, Thurnham-adjusted*†	0·9	0·1, 6·4	0·9	0·2, 3·4	0·6	0·1, 4·0	1·2	0·3, 4·7	0·9	0·3, 2·7
Anaemia with ferritin, Thurnham-adjusted and/or sTfR, no adjustment* ‡	4·6	1·9, 10·5	3·7	1·9, 7·0	3·9	1·9, 8·0	4·1	1·9, 8·3	4·0	2·4, 6·6
Vitamin A deficiency (mild)	0·0	–	0·8	0·2, 3·0	0·5	0·1, 3·4	0·5	0·1, 3·8	0·5	0·1, 2·1
Vitamin A deficiency (severe)	0·0	–	0·0	–	0·0	–	0·0	–	0·0	–
Vitamin B_12_ deficiency	0·0	–	0·0	–	0·0	–	0·0	–	0·0	–
Vitamin D insufficiency	8·8	4·8, 15·7	4·7	2·7, 8·2	1·2†††	0·3, 4·6	11·0‡‡	7·2, 16·4	6·0	4·0, 9·0
Vitamin D deficiency	0·0	–	0·0	–	0·0	–	0·0	–	0·0	–
Zn deficiency	3·4	1·3, 8·9	5·5‡	3·2, 9·3	8·1†† ‡‡	4·8, 13·3	1·5	0·5, 4·7	4·9	3·0, 7·7
Overall (0·5–12·9 years)										
Anaemia§	16·0	12·5, 20·3	13·8	11·6, 16·4	14·3	11·7, 17·4	14·7	12·0, 18·0	14·5	12·6, 16·7
Overall (4·0–12·9 years)										
Fe deficiency (by ferritin, Thurnham-adjusted)||	5·5	3·2, 9·3	8·6	6·2, 11·8	7·9	5·3, 11·7	7·3	4·9, 10·8	7·6	5·7, 10·1
Fe deficiency erythropoiesis (by sTfR)¶	20·8	15·4, 27·4	21·9	18·0, 26·5	23·1	18·5, 28·4	20·0	15·7, 25·2	21·6	18·3, 25·2
Fe deficiency, all stages**	23·4	17·7, 30·1	25·7	21·5, 30·4	26·4	21·6, 32·0	23·4	18·8, 28·8	25·0	21·5, 28·8
Fe deficiency anaemia										
Anaemia with low ferritin, Thurnham-adjusted*†	0·7	0·1, 4·7	1·3	0·6, 2·8	0·7	0·2, 2·5	1·5	0·6, 3·6	1·1	0·5, 2·3
Anaemia with ferritin, Thurnham-adjusted and/or sTfR, no adjustment*‡	4·3	2·1, 8·5	4·1	2·6, 6·5	3·7	2·1, 6·5	4·7	2·7, 7·8	4·2	2·8, 6·1
Vitamin A deficiency (mild)	0·0	–	1·1	0·4, 3·0	0·4	0·0, 2·4	1·2	0·4, 3·7	0·8	0·3, 2·0
Vitamin A deficiency (severe)	0·0	–	0·0	–	0·0	–	0·0	–	0·0	–
Vitamin B_12_ deficiency	0·0	–	0·0	–	0·0	–	0·0	–	0·0	–
Vitamin D insufficiency	7·9*	4·7, 13·0	3·7	2·2, 6·2	1·4†††	0·5, 3·4	8·9	6·0, 13·0	5·0	3·5, 7·2
Vitamin D deficiency	0·0	–	0·0	–	0·0	–	0·0	–	0·0	–
Zn deficiency	2·7	1·1, 6·6	4·2	2·5, 6·9	6·0††	3·7, 9·8	1·3	0·5, 3·5	3·7	2·4, 5·8

* *P* < 0 05: significant difference between urban and rural children based on complex samples Pearson Chi-Square.

† *P* < 0·05,

†† *P* < 0·01,

††† *P* < 0·001: significant difference between boys and girls based on complex samples Pearson Chi-Square.

‡ *P* < 0·05,

‡‡ *P* < 0·01,

‡‡‡ *P* < 0·001: significance of trend of prevalence of biochemical status with age group in each residence and sex.

§ Anaemia, Hb concentrations <110 g/l in children aged 0·5 to <5 years, <115 g/l in children aged 5–11 years and <120 g/l in children aged 12–12·9 years.

|| Fe deficiency by ferritin (Thurnham-adjusted), serum ferritin <12 μg/l in children aged <5 years and <15 μg/l in children aged ≥5 years.

¶ Fe deficiency erythropoiesis by sTfR, sTfR: soluble transferrin receptor >8·3 mg/l.

** Fe deficiency by ferritin (Thurnham-adjusted) below cut-offs and/or sTfR > 8·3 mg/l.

*† Fe deficiency anaemia, Hb below cut-offs with ferritin (Thurnham-adjusted) below cut-offs.

*‡ Fe deficiency anaemia, Hb below cut-offs with Fe deficiency by ferritin (Thurnham-adjusted) below cut-offs and/or sTfR > 8·3 mg/l.

Vitamin A deficiency (mild), serum retinol between 0·35 and 0·7 µmol/l; vitamin A deficiency (severe), serum retinol <0·35 µmol/l.

Vitamin D insufficiency, serum 25-hydroxyvitamin D between 25-<50 nmol/l; vitamin D deficiency, serum 25-hydroxyvitamin D < 25 nmol/l.

Vitamin B_12_ deficiency, serum vitamin B_12_ < 150 pmol/l.

Zn deficiency, plasma Zn <70 *μ*g/dl in girls aged ≥10 years, <74 *μ*g/dl in boys aged ≥10 years, < 65 *μ*g/dl in children aged <10 years.

Fe deficiency among the younger age groups (<4 years old) could not be assessed due to blood collection constraints. Table 4 presents the prevalence of Fe deficiency among children aged over 4 years, where the prevalence of Fe deficiency based on ferritin was 11·9 % and 6·0 % among children in the age groups of 4–6·9 and 7–12·9 years, respectively. However, the prevalence of Fe deficiency erythropoiesis (i.e. by sTfR) was about 2–3 times higher, at 24·3 % and 20·5 %, for the respective age groups. Consequently, the overall prevalence of all stages of Fe deficiency (29·8 % and 23·1 %) was much higher than the prevalence of anaemia (11·5 % and 6·5 %) for children aged 4–6·9 and 7–12·9 years, respectively. There were no statistical differences in the prevalence of anaemia, Fe deficiency or Fe deficiency anaemia between urban and rural areas or by sex for these two age groups. Lastly, among children who were anaemic, Fe deficiency (all stages) contributed 40·9 % and 61·5 % of anaemia in the respective age groups.

The situation of other key micronutrients, namely, vitamin A, vitamin D and Zn, is shown in Table 4. Overall, only 0·8 % of the children had mild vitamin A deficiency, 5 % had vitamin D insufficiency and 3·7 % had Zn deficiency. Borderline or subclinical vitamin A deficiency (serum retinol 0·7–1·05 µmol/l) was 10 % and 13 % in urban and rural areas, respectively (data not shown). None of the children had severe vitamin A deficiency or vitamin B_12_ and vitamin D deficiencies. Nonetheless, a significant difference in vitamin D insufficiency between urban and rural areas was found among children aged 4–6·9 years, whereas significant differences in both vitamin D insufficiency and Zn deficiency by sex were found in children aged 7–12·9 years.

Trends in the prevalence of micronutrient deficiencies were also analysed for the age groups of 4–6·9, 7–9·9 and 10–12·9 years (data not shown). There was a significant downward trend by age group in Fe deficiency based on ferritin (corrected) in the urban area and among boys, as well as a significant downward trend for all stages of Fe deficiency among girls (*P*_trend_ <0·05). Significantly upward trends in vitamin D insufficiency by age group were found in the rural area (*P*_trend_ <0·01) and among girls (*P*_trend_ <0·001). Similarly, a significant upward trend in the prevalence of Zn deficiency by age group was observed in both urban (*P*_trend_ <0·05) and rural areas (*P*_trend_ <0·001) as well as among boys (*P*_trend_ <0·001).

## Discussion

The nationally representative SEANUTS II study in Thailand revealed that triple forms of malnutrition – overnutrition, undernutrition and micronutrient deficiencies – are coexisting among rural and urban Thai children. Overall stunting prevalence is generally low, especially in children older than 4 years of age. Wasting/thinness is indeed more prevalent among older children, but it is still low in terms of being of public health significance. However, most striking is the very high prevalence of overweight and obesity among children aged 7–12·9 years old. Energy and macronutrient intakes as percentage of energy intake are in line with recommended intakes, although mean protein intake (g/d) appears to be much higher than the daily recommended allowances across all ages. The risks of having inadequate intakes of some key micronutrients, that is, Ca, Fe, Zn, vitamin A, vitamin C and vitamin D, are high (>50 %). These estimates may be inaccurate since the data were not corrected for within-person variation (based on one 24-h recall). In addition, anaemia among infants and toddlers and Fe deficiency among older children are persistent challenges. Deficiencies of other micronutrients are minimal, though subclinical vitamin A deficiency and vitamin D insufficiency are still observed. Some discrepancies in the prevalence of Zn deficiency and vitamin D insufficiency by areas of residence and sex are noted among older age children (7–12·9 years old). We did not adjust serum retinol for inflammation. Hence, the prevalence of subclinical vitamin A deficiency may be overestimated.

The present survey showed that older children, specifically those around primary school age (7–12·9 years old), are facing both ends of the double burden malnutrition spectrum (thinness and overweight/obesity), which deserves much more attention. Time trends are important to place the findings in perspective. In Thailand and other Asian countries, the prevalence of stunting and wasting among young children persists but has declined in both numbers and severity over time. Overweight and obesity, on the other hand, have rapidly increased among both children and adults^(9,36,37)^.

The prevalence of stunting and wasting, especially among rural children, was lower than that reported a decade ago (SEANUTS I)^(11)^. The increase in obesity prevalence among rural children is catching up with that of their urban counterparts, while overweight and obesity in urban areas have not shown any decline. This evidence indicates that the pattern of urban–rural difference in the double burden of malnutrition is diminishing. This situation cannot be treated with complacency, as the rapid rise of overweight and obesity is becoming more prominent, while declines in the stunting and wasting among young children are not yet in a favourable place.

In this study, stunting was highest in boys under 4 years, and obesity was also the highest in boys from 0·5 to 12 years old. Data from other national-scale surveys also have shown a higher trend in the prevalence of stunting and obesity in boys compared with girls. Among a survey conducted in 2014 among children aged 1–5 years, the prevalence of stunting in boys was 9·1 %, while it was 6·0 % in girls^(9)^. The same trend was found in the MICS survey in 2019, where the prevalence of stunting was 15·2 % in boys aged 0–5 years compared with 11·4 % in girls^(7)^. With regard to obesity, the prevalence for boys was higher than the prevalence for girls in 2014 (10 % *v*. 3·5 % in children aged 6–11 years and 11·9 % *v*. 5·6 % in children aged 12–14 years)^(9)^. Similarly, findings from the Global School-based Student Health Survey conducted in 2021 among children and adolescents in grades 7–12 showed a higher prevalence of obesity in boys compared with girls (8·1 % *v*. 5·1 %)^(38)^. These findings warrant further investigations to clarify whether they were the results of a cultural phenomenon or a genetic one.

On average, the energy intake of children met the estimated energy requirement. Protein intake as % energy was around 15 % (except for the infant group), but mean intakes per day were very high relative to EAR or even recommended daily allowances for protein. While an upper limit for protein intake has not been established, EFSA recommends that protein intake should not exceed 20 % of total energy intake^(39)^. Too high protein intake during the first 2 years of life, and possibly into later childhood, can increase the risk of obesity and related diseases in later life^(40)^. An emerging issue is low dietary fibre consumption (2–7 g/d), which is about half of the recommended daily intake (age in years plus 5 g/d)^(23)^. Although consumption in high amounts can compromise the absorption of some essential nutrients, the beneficial effects of dietary fibre on gut health and beyond (e.g. anti-inflammatory effects) are associated with lower risks for chronic diseases^(41)^. Promotion of adequate dietary fibre intakes among children, both in terms of quantity and quality (solubility, fermentability and viscosity), is advisable.

Thailand’s economic and demographic development and transformation began to influence rural consumers more in the 1990s and 2000s through the expansion of modern retail outlets into regional centres and towns^(42)^. Consequently, the food environment and food system need greater attention in this rapidly developing society^(43)^. Increases in urbanisation and modernisation of food retail systems, whereby processed and non-nutritious energy-dense foods have become more accessible even in rural areas, can adversely contribute to the diets and health of populations in Thailand and other developing countries^(44)^. Over time, there has been a substantial change in food consumption from traditional Thai diets and food patterns (mainly grain/plant-based) to diets high in animal protein, dairy products and the inclusion of processed foods and refined grains^(45)^. Additionally, the Thai population consume more Western fast food and sweetened beverages^(46,47)^.

This study revealed that the risks of inadequate intakes of key micronutrients are very high, especially among older children. This risk among children aged 7–12·9 years could be due to their being more independent in choosing their own foods, especially outside the home, which could compromise food intake quantity and quality. Moreover, older children may be more concerned about their body image and possibly underreport their dietary intakes^(48)^. In SEANUTS I, the mean intakes of several micronutrients and intake trends by age are similar to those found in the SEANUTS II survey. The risk of inadequate intakes of micronutrients was over 50 %, after correcting for intra-individual variability^(11)^. The magnitude of risks of having inadequate intakes in the current study could be biased as it was based on a 1-d 24-h intake report and without accounting for intra-individual variability (Table 2). Furthermore, the high risk of inadequate Ca intake, despite the widely implemented school milk programme, deserves closer examination in terms of programme coverage and effectiveness.

Anaemia among infants and young children was especially high (56·6 % and 35·2 % for infants and 1 to <4 years, respectively). A 2018 survey conducted by the Department of Health in four geographical regions of Thailand also reported high prevalence of anaemia in infants and toddlers (43·1 and 44·5 %) and preschool children (27·3 %)^(49)^. Unfortunately, the contribution of Fe deficiency in these young children was not ascertained. As found in older age groups, Fe deficiency remains a significant cause of anaemia. The much higher prevalence of Fe deficiency without anaemia than for prevalence of anaemia warrants concern. The global analysis of causes of anaemia showed that Fe deficiency and haemoglobinopathy/haemolytic anaemia were the two most important causes in the aetiology of anaemia^(50)^. In Southeast Asia, carriers of underlying genetic Hb disorders/haemoglobinopathy are highly prevalent in some countries, including Thailand^(51)^. Given similar dietary patterns within the same population, some of these genetic carriers may also be Fe deficient. Since there is a dearth of data on Fe profiles among these genetic carriers, interpretation of Fe deficiency needs further investigation.

Vitamin D insufficiency in SEANUTS II was much less prevalent than that reported in SEANUTS I^(52)^ (5·0 % *v*. 31·7 %, respectively). Although different assay methods were used (chemiluminescence immunoassay [LIAISON^®^, Diasorin, Inc.] in SEANUTS I *v*. LC–MS/MS for SEANUTS II), a prior study has shown only a small difference between these methods^(53)^. Consequently, the difference in assay methods is unlikely to account for the large differences in the prevalence between the two surveys. In fact, the findings from SEANUTS I triggered actions by both public and private sectors to conduct campaigns promoting outdoor physical activities^(54)^, as well as the development of vitamin D-fortified food products and their marketing.

Better vitamin D status among rural children compared with urban children and better vitamin D status among boys than girls aged 7–12·9 years were shown. These findings may be associated with being more physically active and exposed to outdoor activities, since de novo synthesis by sun exposure is of utmost importance for adequate vitamin D status^(55)^. Further analysis on physical activity and UV exposure will be performed to determine whether these factors effect vitamin D status.

Despite the overall low prevalence of Zn deficiency, boys were significantly worse off compared with girls in the oldest age groups, which included children entering puberty. During this time, Zn requirements are high to meet the needs of incremental growth and the building of muscle mass, as well as to compensate for endogenous Zn losses in semen and menses, in addition to urinary, sweat and integumental losses^(56)^. Inadequate dietary intakes coupled with higher requirements in boys may explain the significant difference in Zn deficiency by sex.

SEANUTS II was planned to be conducted among a nationally representative sample of children. However, the first wave of the COVID-19 pandemic in Thailand emerged in early 2020, after the survey was implemented and data collection among 477 children was completed. As a result, a large part of data collection (86·3 % of children) was completed during July to December 2020. A rapid assessment conducted in May 2020 reported no anticipated impact on food security and nutrition in the short term^(57)^. Further data analyses to confirm the possible impacts of the COVID-19 pandemic on our findings are reported elsewhere^(58)^.

Despite the careful survey design and assessment protocol, a couple of limitations should be noted. For dietary assessment, only 1-d 24-h recall was feasible, which may be biased and limit the generalisability of the risks of inadequate nutrient intakes. The intra-individual variability correction was not undertaken in the current study because we did not perform the second-day 24-h recall, and the available data from another survey were 10 years ago and used a different nutrient database. Genetic-related haemoglobinopathy was not included in this report, which may play a role in terms of anaemia prevalence. Further analysis could provide better evidence of the contributions of Fe deficiency and haemoglobinopathy to anaemia in Thai children.

We found discrepancies in findings based on dietary assessment and biomarkers, which could be explained by differences in the stages of measurement. Dietary assessment tends to be a useful index of the exposure stage of the nutrients as consumed through foods, but it does not necessarily reflect nutritional status such as those of biomarkers or biochemical measures based on metabolism or physiological processes^(59,60)^. In public health practice, nutritional status based on biochemical measures is used to demonstrate a public health concern. Considering the context of public health and its implications, inadequate nutrient intakes based on a single 24-h recall serve as an early warning sign that warrants a reinvestigation as to whether or not the insufficient intake of the nutrient in question is relevant.

### Conclusions

Findings from this study highlight that Thailand continues to experience a triple burden of malnutrition, with a clear shift to the manifestation of overweight and obesity as public health problems. Hence, there is a need for periodic monitoring of child nutritional status and the drivers of malnutrition to inform policy-making and the design of effective programmes to tackle these drivers. These drivers may include early life nutrition, dietary diversity, food environments and socio-economic factors. For instance, the prevalence of stunting and anaemia was still high among Thai children aged 1–3·9 years, which reflects a gap in promoting healthy diets during this transitional period, from being cared for by caregivers/family to day care centres.

In this context, current efforts should be strengthened to promote proper infant and young child feeding practices within the government’s priority Miracle of 1000 d policy. Micronutrients are necessary for optimal growth, bone health, immunity and cognitive development in children. Hence, food-based dietary guidelines coupled with effective behavioural change communication to increase the consumption of micronutrient-dense foods should be considered. Continued attention focusing on all forms of malnutrition among older age children through the primary school is also warranted. The quality of meals served at home, within the school food environment and lifestyle factors (especially among adolescents) must be considered as they impact on the nutritional well-being of preschool and school-age children. Finally, holistic public policies and programmes involving the food, health, education and social protection sectors that are designed with community inputs are needed to address the broader range of malnutrition drivers and thus overcome the nutritional challenges of Thai children.

## Supporting information

Pongcharoen et al. supplementary material Pongcharoen et al. supplementary material

## Supplementary material

For supplementary material accompanying this paper visit https://doi.org/10.1017/S1368980024000053.

## References

[1] Kelly M (2016) The nutrition transition in developing Asia: Dietary change, drivers and health impacts. In Eating, Drinking: Surviving SpringerBriefs in Global Understanding, pp. 83–90 [ P Jackson , W Spiess & F Sultana , editors]. Cham: Springer.

[2] Development Initiatives (2020) Global Nutrition Report: Action on Equity to End Malnutrition. Bristol: Development Initiatives Poverty Research Ltd.

[3] Rachmi CN , Li M & Baur LA (2018) The double burden of malnutrition in association of South East Asian Nations (ASEAN) countries: a comprehensive review of the literature. Asia Pac J Clin Nutr 27, 736–755. 30045417 10.6133/apjcn.062018.02

[4] Roos N , Ponce MC , Doak CM et al. (2019) Micronutrient status of populations and preventive nutrition interventions in South East Asia. Matern Child Health J 23, 29–45. 30506126 10.1007/s10995-018-2639-2

[5] Stevens GA , Paciorek CJ , Flores-Urrutia MC et al. (2022) National, regional, and global estimates of anaemia by severity in women and children for 2000-19: a pooled analysis of population-representative data. Lancet Glob Health 10, e627–e639. 35427520 10.1016/S2214-109X(22)00084-5 PMC9023869

[6] National Statistical Office & Nations Children’s Fund United (2016) Thailand Multiple Indicator Cluster Survey 2015–2016, Final Report. Bangkok: NSO and UNICEF.

[7] National Statistical Office (2020) Thailand Multiple Indicator Cluster Survey 2019, Survey Findings Report. Bangkok: National Statistical Office of Thailand.

[8] Aekplakorn W (2011) Report on the 4th Thai National Health Examination Survey (NHES IV), 2008–2009: Child health. Nonthaburi, Thailand: Health Systems Research Institute.

[9] Aekplakorn W (2014) Report on the 5th Thai National Health Examination Survey (NHES V), 2014: Child health. Nonthaburi, Thailand: Health Systems Research Institute.

[10] Schaafsma A , Deurenberg P , Calame W et al. (2013) Design of the South East Asian Nutrition Survey (SEANUTS): a four-country multistage cluster design study. Br J Nutr 110, S2–10. 24016763 10.1017/S0007114513002067

[11] Rojroongwasinkul N , Kijboonchoo K , Wimonpeerapattana W et al. (2013) SEANUTS: the nutritional status and dietary intakes of 0.5–12-year-old Thai children. Br J Nutr 110, S36–44. 24016765 10.1017/S0007114513002110

[12] Department of Health (2018) Implementation Plan for the Miracle of 1,000 Days Policy. Nonthaburi, Thailand: Department of Health, Ministry of Public Health.

[13] Tan SL , Poh BK , Sekartini R et al. (2024) South East Asian Nutrition Survey (SEANUTS) II, multi-country evaluation of nutrition and lifestyle indicators in children aged 12 years and below: rationale and design. *Public Health Nutr*. In Review. 10.1017/S1368980024000910 PMC11617418 38639132

[14] Gorstein J , Sullivan K , Parvanta I et al. (2007) Indicators and Methods for Cross-Sectional Surveys of Vitamin and Mineral Status of Populations. Ottawa/Atlanta: The Micronutrient Initiative and the Centers for Disease Control and Prevention.

[15] World Health Organization (2008) Training course on child growth assessment. WHO child growth standards. Geneva: World Health Organization.

[16] World Health Organization (2006) WHO child growth standards. Available at https://www.who.int/tools/child-growth-standards/standards (accessed December 2021).

[17] World Health Organization (2007) WHO growth reference data for children 5–19 years. Available at https://www.who.int/tools/growth-reference-data-for-5to19-years/indicators (accessed December 2021).

[18] World Health Organization (2010) WHO Anthro for personal computers, version 3.2.2, 2011: Software for assessing growth and development of the world’s children. Geneva: World Health Organization.

[19] World Health Organization (2009) WHO AnthroPlus for personal computers manual: Software for assessing growth of the world’s children and adolescents. Geneva: World Health Organization.

[20] Briefel RR , Kalb LM , Condon E et al. (2010) The feeding infants and toddlers study 2008: study design and methods. J Am Diet Assoc 110, S16–26. 21092765 10.1016/j.jada.2010.09.005

[21] Institute of Nutrition (2013) INMUCAL-Nutrients V 3.1 Software. Nakhon Pathom: Institute of Nutrition, Mahidol University.

[22] Institute of Medicine (2000) Dietary Reference Intakes: Applications in Dietary Assessment. Washington, DC: The National Academies Press. 25057725

[23] Bureau of Nutrition (2020) Dietary Reference Intake for Thais 2020. Nonthaburi, Thailand: Department of Health, Ministry of Public Health.

[24] Allen LH , Carriquiry AL & Murphy SP (2020) Perspective: proposed harmonized nutrient reference values for populations. Adv Nutr 11, 469–483. 31701998 10.1093/advances/nmz096 PMC7231601

[25] Mendez MA , Popkin BM , Buckland G et al. (2011) Alternative methods of accounting for underreporting and overreporting when measuring dietary intake-obesity relations. Am J Epidemiol 173, 448–458. 21242302 10.1093/aje/kwq380 PMC3139974

[26] Food and Agriculture Organization of the United Nations/World Health Organization/United Nations University (2004) Human Energy Requirements: Report of a Joint FAO/WHO/UNU Expert Consultation, 17–24 October 2001. Rome: Food and Agriculture Organization of the United Nations.

[27] International Zinc Nutrition Consultative Group (2012) Assessing Population Zinc Status with Serum Zinc Concentration. IZiNCG Technical Brief No. 02, 2nd ed. Davis (CA): IZinCG.

[28] World Health Organization (2011) Haemoglobin concentrations for the diagnosis of anaemia and assessment of severity. In Vitamin and Mineral Nutrition Information System. Geneva: World Health Organization.

[29] Thurnham DI , Northrop-Clewes CA & Knowles J (2015) The use of adjustment factors to address the impact of inflammation on vitamin A and iron status in humans. J Nutr 145, 1137S–1143S. 25833890 10.3945/jn.114.194712 PMC4410494

[30] World Health Organization & Centers for Disease Control and Prevention (2007) Assessing the *Iron Status of Populations*. Geneva: WHO Press.

[31] Phiri KS , Calis JC , Siyasiya A et al. (2009) New cut-off values for ferritin and soluble transferrin receptor for the assessment of iron deficiency in children in a high infection pressure area. J Clin Pathol 62, 1103–1106. 19946096 10.1136/jcp.2009.066498 PMC2776133

[32] King JC , Brown KH , Gibson RS et al. (2015) Biomarkers of nutrition for development (BOND)-zinc review. J Nutr 146, 858S–885S. 26962190 10.3945/jn.115.220079 PMC4807640

[33] de Pee S & Dary O (2002) Biochemical indicators of vitamin A deficiency: serum retinol and serum retinol binding protein. J Nutr 132, 2895S–2901S. 12221267 10.1093/jn/132.9.2895S

[34] de Benoist B (2008) Conclusions of a WHO technical consultation on folate and vitamin B12 deficiencies. Food Nutr Bull 29, S238–244. 18709899 10.1177/15648265080292S129

[35] Misra M , Pacaud D , Petryk A et al. (2008) Vitamin D deficiency in children and its management: review of current knowledge and recommendations. Pediatrics 122, 398–417. 18676559 10.1542/peds.2007-1894

[36] Winichagoon P (2015) Transition of maternal and child nutrition in Asia: implications for public health. Curr Opin Clin Nutr Metab Care 18, 312–317. 25807348 10.1097/MCO.0000000000000158

[37] NCD Risk Factor Collaboration (NCD-RisC) (2017) Worldwide trends in body-mass index, underweight, overweight, and obesity from 1975 to 2016: a pooled analysis of 2416 population-based measurement studies in 128.9 million children, adolescents, and adults. Lancet 390, 2627–2642. 29029897 10.1016/S0140-6736(17)32129-3 PMC5735219

[38] Department of Health & Ministry of Public Hleath (2022) Thailand Global School-based Student Health Survey, 2021: GSHS. Pathum Thani: Department of Health, Ministry of Public Health.

[39] EFSA & Panel on Dietetic Products, Nutrition and Allergies (NDA) (2012) Scientific opinion on dietary reference values for protein. EFSA J 10, 66.

[40] Koletzko B , Demmelmair H , Grote V et al. (2016) High protein intake in young children and increased weight gain and obesity risk. Am J Clin Nutr 103, 303–304. 26791192 10.3945/ajcn.115.128009

[41] Hojsak I , Benninga MA , Hauser B et al. (2022) Benefits of dietary fibre for children in health and disease. Arch Dis Child 107, 973–979. 35277379 10.1136/archdischild-2021-323571 PMC9606532

[42] Ngamprasertkit S (2016) GAIN Report Number TH 6160: Thailand Retail Foods 2016. Global Agricultural Information Network: USDA Foreign Agricultural Service. Available at https://agriexchange.apeda.gov.in/MarketReport/Reports/Retail_Foods_Bangkok_Thailand_12-29-2016.pdf (accessed February 2022).

[43] Hawkes C , Ruel MT , Salm L et al. (2020) Double-duty actions: seizing programme and policy opportunities to address malnutrition in all its forms. Lancet 395, 142–155. 31852603 10.1016/S0140-6736(19)32506-1

[44] Baker P & Friel S (2014) Processed foods and the nutrition transition: evidence from Asia. Obes Rev 15, 564–577. 24735161 10.1111/obr.12174

[45] Pingali P (2007) Westernization of Asian diets and the transformation of food systems: implications for research and policy. Food Policy 32, 281–298.

[46] National Statistical Office (2014) The 2013 Food Consumption Behavior Survey. Bangkok: National Statistical Office.

[47] National Statistical Office (2018) The 2017 Food Consumption Behavior Survey. Bangkok: National Statistical Office.

[48] Song L , Zhang Y , Chen T et al. (2022) Association of body perception and dietary weight management behaviours among children and adolescents aged 6–17 years in China: cross-sectional study using CHNS (2015). BMC Public Health 22, 175. 35081917 10.1186/s12889-022-12574-6 PMC8790848

[49] Bureau of Nutrition (2021) Research report: Study on the factors affecting the growth and the promotion model of the full potential growth among childhood Thai children. Nonthaburi, Thailand: Department of Health, Ministry of Public Health.

[50] Safiri S , Kolahi AA , Noori M et al. (2021) Burden of anemia and its underlying causes in 204 countries and territories, 1990–2019: results from the Global Burden of Disease Study 2019. J Hematol Oncol 14, 185. 34736513 10.1186/s13045-021-01202-2 PMC8567696

[51] Thurlow RA , Winichagoon P , Green T et al. (2005) Only a small proportion of anemia in northeast Thai school children is associated with iron deficiency. Am J Clin Nutr 82, 380–387. 16087982 10.1093/ajcn.82.2.380

[52] Poh BK , Rojroongwasinkul N , Nguyen BK et al. (2016) 25-hydroxy-vitamin D demography and the risk of vitamin D insufficiency in the South East Asian Nutrition Surveys (SEANUTS). Asia Pac J Clin Nutr 25, 538–548. 27440689 10.6133/apjcn.092015.02

[53] Moon HW , Cho JH , Hur M et al. (2012) Comparison of four current 25-hydroxyvitamin D assays. Clin Biochem 45, 326–330. 22244986 10.1016/j.clinbiochem.2011.12.025

[54] Topothai T , Khamput T , Kamonrungsan J et al. (2019) Lessons learnt from developing the 2018–2030 national physical activity plan in Thailand. J Health Syst Res 13, 442–456.

[55] Wacker M & Holick MF (2013) Sunlight and Vitamin D: a global perspective for health. Dermatoendocrinol 5, 51–108. 24494042 10.4161/derm.24494 PMC3897598

[56] International Zinc Nutrition Consultative Group, Brown KH , Rivera JA et al. (2004) International zinc nutrition consultative group (IZiNCG) technical document #1. Assessment of the risk of zinc deficiency in populations and options for its control. Food Nutr Bull 25, S99–203. 18046856

[57] Policy Management Oxford & Nations Thailand United (2020) Social Impact Assessment of COVID-19 in Thailand. Oxford: Oxford Policy Management.

[58] Geurts J , Singh-Povel C , Lee ST et al. The impact of the COVID-19 pandemic in Southeast Asia: insights from the SEANUTS II study. *Public Health Nutr*.

[59] Gibson R (2005) Principles of nutritional assessment, 2nd ed. New York, NY: Oxford University Press, Inc.

[60] Raiten DJ , Combs GF , Steiber AL et al. (2021) Perspective: nutritional status as a biological variable (NABV): integrating nutrition science into basic and clinical research and care. Adv Nutr 12, 1599–1609. 34009250 10.1093/advances/nmab046 PMC8483963

