# Supplementary material for: South East Asian Nutrition Surveys II (SEANUTS II) Thailand: triple burden of malnutrition among Thai children aged 6 months to 12 years
Source: Public Health Nutr. 2024 Jan 22;27(1):e152. doi: 10.1017/S1368980024000053 (PMC11617422; doi:10.1017/S1368980024000053)
Supplement: Pongcharoen et al. supplementary material [file S1368980024000053sup001.docx]

**Supplementary Table 1** Weighted data of socio-demographic characteristics of the children by residence

|  | Urban | | Rural | | All | |
| --- | --- | --- | --- | --- | --- | --- |
| Variables | Mean | SE | Mean | SE | Mean | SE |
| Household monthly income (USD)^§^ | 646.8^*^ | 17.4 | 514.8 | 10.7 | 556.2 | 9.2 |
| Household monthly food expenditure (USD)^§^ | 244.2^*^ | 5.5 | 199.5 | 3.2 | 213.5 | 2.8 |
| Mother’s age | 34.6 | 0.3 | 35.1 | 0.2 | 35.0 | 0.2 |
| Father’s age | 37.9 | 0.3 | 38.5 | 0.2 | 38.3 | 0.2 |
|  |  | |  | |  | |
| Mother’s education | % | | % | | % | |
| No-schooling/primary school | 13.4^*^ | | 23.8 | | 20.5 | |
| Secondary school (including high school) | 61.8 | | 56.7 | | 58.3 | |
| Tertiary school (including diploma, bachelor, master, doctor, college, university) | 24.8 | | 19.5 | | 21.2 | |
| Father’s education |  | |  | |  | |
| No-schooling/primary school | 23.7^*^ | | 28.5 | | 27.0 | |
| Secondary school (including high school) | 55.1 | | 56.5 | | 56.1 | |
| Tertiary school (including diploma, bachelor, master, doctor, college, university) | 21.2 | | 15.0 | | 17.0 | |

^*^*P*<0.001: significant difference between the urban and rural children.

^§^1 US$ = 31.29 Thai Baht (June 21, 2021).

**Supplementary Table 2** Weighted data of anthropometric characteristics of the children by age group, residence, and sex

| Variables | Residence | | | | Sex | | | | All | |
| --- | --- | --- | --- | --- | --- | --- | --- | --- | --- | --- |
|  | Urban | | Rural | | Boys | | Girls | |  |  |
|  | Mean | SE | Mean | SE | Mean | SE | Mean | SE | Mean | SE |
| 0.5-0.9 years |  |  |  |  |  |  |  |  |  |  |
| Weight (kg) | 8.3 | 0.1 | 8.5 | 0.1 | 8.8^†††^ | 0.1 | 8.1 | 0.1 | 8.4 | 0.1 |
| Height (cm) | 69.6 | 0.3 | 70.0 | 0.2 | 70.7^†††^ | 0.3 | 69.1 | 0.3 | 69.8 | 0.2 |
|  |  |  |  |  |  |  |  |  |  |  |
| 1.0-3.9 years |  |  |  |  |  |  |  |  |  |  |
| Weight (kg) | 13.5 | 0.2 | 13.4 | 0.1 | 13.8^†††^ | 0.1 | 13.1 | 0.1 | 13.4 | 0.1 |
| Height (cm) | 90.4 | 0.5 | 90.0 | 0.3 | 90.7^†††^ | 0.4 | 89.6 | 0.4 | 90.2 | 0.3 |
| BMI (kg/m^2^) | 16.4 | 0.1 | 16.4 | 0.1 | 16.6^††^ | 0.1 | 16.1 | 0.1 | 16.4 | 0.1 |
|  |  |  |  |  |  |  |  |  |  |  |
| 4.0-6.9 years |  |  |  |  |  |  |  |  |  |  |
| Weight (kg) | 19.3^*^ | 0.3 | 20.5 | 0.3 | 20.8^††^ | 0.4 | 19.3 | 0.3 | 20.1 | 0.3 |
| Height (cm) | 111.3 | 0.5 | 112.5 | 0.4 | 112.9^†††^ | 0.4 | 111.1 | 0.4 | 112.1 | 0.3 |
|  |  |  |  |  |  |  |  |  |  |  |
| 7.0-12.9 years |  |  |  |  |  |  |  |  |  |  |
| Weight (kg) | 35.9 | 0.7 | 34.8 | 0.5 | 35.0 | 0.6 | 35.4 | 0.6 | 35.2 | 0.4 |
| Height (cm) | 137.9^*^ | 0.7 | 137.3 | 0.5 | 136.9^†^ | 0.5 | 138.0 | 0.6 | 137.5 | 0.4 |
| BMI (kg/m^2^) | 18.3 | 0.2 | 18.0 | 0.2 | 18.1 | 0.2 | 18.0 | 0.2 | 18.1 | 0.1 |
|  |  |  |  |  |  |  |  |  |  |  |
| <5 years (0.5-4.9 years) |  |  |  |  |  |  |  |  |  |  |
| Weight (kg) | 13.5 | 0.2 | 13.4 | 0.1 | 13.9^†††^ | 0.2 | 13.0 | 0.1 | 13.4 | 0.1 |
| Height (cm) | 89.9 | 0.6 | 89.8 | 0.4 | 90.7^†††^ | 0.5 | 88.9 | 0.5 | 89.9 | 0.3 |

^*^*P*<0.05: significant difference between the urban and rural children based on complex samples ANCOVA after correcting for age.

^†^*P*<0.05, ^††^*P*<0.01, ^†††^*P*<0.001: significant difference between boys and girls based on complex samples ANCOVA after correcting for age**.**

**Supplementary Table 3** Weighted anthropometric nutritional status indices (z-score) by age group, residence, and sex

| Variables | Residence | | | | Sex | | | | All | |
| --- | --- | --- | --- | --- | --- | --- | --- | --- | --- | --- |
|  | Urban | | Rural | | Boys | | Girls | |  | |
|  | Mean | SE | Mean | SE | Mean | SE | Mean | SE | Mean | SE |
| 0.5-0.9 years |  |  |  |  |  |  |  |  |  |  |
| HAZ | -0.66 | 0.09 | -0.64 | 0.08 | -0.69 | 0.08 | -0.60 | 0.09 | -0.65 | 0.06 |
| WAZ | -0.33 | 0.09 | -0.25 | 0.08 | -0.25 | 0.09 | -0.30 | 0.09 | -0.28 | 0.06 |
| WHZ | 0.08 | 0.09 | 0.13 | 0.08 | 0.20 | 0.09 | 0.03 | 0.08 | 0.12 | 0.06 |
| BAZ | 0.07 | 0.09 | 0.13 | 0.08 | 0.20 | 0.09 | 0.02 | 0.08 | 0.11 | 0.06 |
|  |  |  |  |  |  |  |  |  |  |  |
| 1.0-3.9 years |  |  |  |  |  |  |  |  |  |  |
| HAZ | -0.33 | 0.06 | -0.41 | 0.04 | -0.40 | 0.05 | -0.37 | 0.04 | -0.38 | 0.03 |
| WAZ | 0.04 | 0.07 | 0.04 | 0.05 | 0.07 | 0.05 | 0.00 | 0.05 | 0.04 | 0.04 |
| WHZ | 0.24 | 0.07 | 0.33 | 0.04 | 0.35 | 0.05 | 0.25 | 0.05 | 0.30 | 0.04 |
| BAZ | 0.29 | 0.07 | 0.39 | 0.04 | 0.41 | 0.05 | 0.30 | 0.05 | 0.36 | 0.04 |
|  |  |  |  |  |  |  |  |  |  |  |
| 4.0-6.9 years |  |  |  |  |  |  |  |  |  |  |
| HAZ | -0.34 | 0.07 | -0.21 | 0.05 | -0.18^†^ | 0.06 | -0.34 | 0.05 | -0.25 | 0.04 |
| WAZ | -0.25 | 0.09 | -0.12 | 0.07 | -0.06 | 0.09 | -0.27 | 0.07 | -0.16 | 0.06 |
| WHZ (4-4.9 y) | 0.09 | 0.11 | 0.03 | 0.09 | 0.07 | 0.11 | 0.03 | 0.10 | 0.05 | 0.07 |
| BAZ | -0.07 | 0.09 | -0.02 | 0.07 | 0.06 | 0.09 | -0.14 | 0.07 | -0.04 | 0.06 |
|  |  |  |  |  |  |  |  |  |  |  |
| 7.0-12.9 years |  |  |  |  |  |  |  |  |  |  |
| HAZ | -0.04^*^ | 0.05 | -0.18 | 0.04 | -0.15^†^ | 0.04 | -0.13 | 0.05 | -0.14 | 0.03 |
| WAZ (7-10 y) | 0.16 | 0.11 | 0.03 | 0.08 | 0.11 | 0.10 | 0.04 | 0.09 | 0.07 | 0.07 |
| BAZ | 0.37^*^ | 0.08 | 0.15 | 0.06 | 0.26 | 0.07 | 0.18 | 0.06 | 0.22 | 0.05 |
|  |  |  |  |  |  |  |  |  |  |  |
| <5 years (0.5-4.9 years) |  |  |  |  |  |  |  |  |  |  |
| HAZ | -0.38 | 0.04 | -0.44 | 0.03 | -0.41 | 0.04 | -0.43 | 0.03 | -0.42 | 0.02 |
| WAZ | -0.07 | 0.05 | -0.07 | 0.04 | -0.03 | 0.04 | -0.11 | 0.04 | -0.07 | 0.03 |
| WHZ | 0.17 | 0.05 | 0.23 | 0.04 | 0.26 | 0.04 | 0.16 | 0.04 | 0.21 | 0.03 |
| BAZ | 0.20 | 0.05 | 0.27 | 0.04 | 0.31^†^ | 0.04 | 0.18 | 0.04 | 0.25 | 0.03 |

HAZ, height-for-age z-scores; WAZ, weight-for-age z-scores; WHZ, weight-for-height z-scores; BAZ, BMI-for-age z-scores.

^*^*P*<0.05: significant difference between the urban and rural children based on complex samples ANCOVA after correcting for age.

^†^*P*<0.05: significant difference between the boys and girls based on complex samples ANCOVA after correcting for age**.**

**Supplementary Table 4** Weighted percentage of stunting, underweight, wasting, thinness, overweight, and obesity by age group, sex, and residence

|  | Residence | | | | Sex | | | |  | |
| --- | --- | --- | --- | --- | --- | --- | --- | --- | --- | --- |
|  | Urban | | Rural | | Boys | | Girls | | All | |
| Variables | % | 95% CI | % | 95% CI | % | 95% CI | % | 95% CI | % | 95% CI |
| 0.5-0.9 years |  |  |  |  |  |  |  |  |  |  |
| Stunting | 7.6 | 3.8, 14.6 | 10.6 | 7.0, 15.7 | 12.0 | 7.8, 18.1 | 7.2 | 3.9, 12.9 | 9.6 | 6.7, 13.5 |
| Underweight | 4.1 | 1.6, 10.6 | 5.2 | 2.8, 9.5 | 3.2 | 1.3, 7.4 | 6.5 | 3.4, 12.0 | 4.9 | 2.9, 8.1 |
| Wasting | 2.0 | 0.5, 7.7 | 1.6 | 0.5, 4.9 | 1.3 | 0.3, 5.0 | 2.2 | 0.7, 6.6 | 1.7 | 0.7, 4.1 |
| Overweight | 1.8 | 0.5, 7.0 | 3.1 | 1.4, 6.8 | 3.2 | 1.3, 7.4 | 2.2 | 0.7, 6.6 | 2.7 | 1.3, 5.3 |
| Obesity | 0.0 | - | 1.4 | 0.5, 4.3 | 1.9 | 0.6, 5.8 | 0.0 | - | 0.9 | 0.3, 2.9 |
|  |  |  |  |  |  |  |  |  |  |  |
| 1.0-3.9 years |  |  |  |  |  |  |  |  |  |  |
| Stunting | 4.5 | 2.8, 7.2 | 7.0 | 5.4, 9.0 | 7.3 | 5.4, 9.6 | 5.0 | 3.5, 7.0 | 6.2 | 5.0, 7.7 |
| Underweight | 3.7 | 2.2, 6.2 | 3.5 | 2.4, 5.0 | 2.5^†^ | 1.5, 4.2 | 4.7 | 3.2, 6.8 | 3.5 | 2.6, 4.8 |
| Wasting | 2.0 | 1.0, 4.0 | 1.5 | 0.9, 2.7 | 1.4 | 0.7, 2.7 | 2.0 | 1.1, 3.5 | 1.7 | 1.1, 2.6 |
| Overweight | 6.8 | 4.6, 10.0 | 6.7 | 5.2, 8.8 | 7.7 | 5.8, 10.3 | 5.6 | 4.0, 7.8 | 6.8 | 5.4, 8.4 |
| Obesity | 4.1 | 2.5, 6.7 | 3.5 | 2.4, 5.0 | 3.5 | 2.3, 5.3 | 3.9 | 2.5, 5.8 | 3.7 | 2.7, 4.9 |
|  |  |  |  |  |  |  |  |  |  |  |
| 4.0-6.9 years |  |  |  |  |  |  |  |  |  |  |
| Stunting | 5.4 | 3.0, 9.4 | 3.5 | 2.2, 5.5 | 4.2 | 2.5, 6.9 | 4.0 | 2.4, 6.5 | 4.1 | 2.8, 5.8 |
| Underweight (4-4.9 y) | 5.4 | 2.4, 11.5 | 5.1 | 2.9, 8.9 | 4.8 | 2.4, 9.4 | 5.6 | 3.1, 10.2 | 5.2 | 3.3, 8.2 |
| Wasting (4-4.9 y) | 2.7 | 0.9, 8.1 | 4.0 | 2.1, 7.5 | 4.2 | 2.0, 8.6 | 2.8 | 1.2, 6.6 | 3.6 | 2.0, 6.2 |
| Thinness (5-6.9 y) | 4.6 | 1.9, 10.9 | 4.5 | 2.5, 8.2 | 5.9 | 3.1, 10.9 | 3.1 | 1.4, 6.5 | 4.6 | 2.8, 7.4 |
| Overweight | 7.6 | 4.5, 12.6 | 8.1 | 5.7, 11.2 | 8.2 | 5.4, 12.2 | 7.6 | 5.1, 11.1 | 7.9 | 6.0, 10.5 |
| Obesity | 5.9 | 3.3, 10.3 | 10.2 | 7.5, 13.7 | 10.1 | 7.0, 14.4 | 7.4 | 5.0, 10.9 | 8.8 | 6.7, 11.5 |
|  |  |  |  |  |  |  |  |  |  |  |
| 7.0-12.9 years |  |  |  |  |  |  |  |  |  |  |
| Stunting | 2.0^*,‡^ | 1.0, 4.2 | 4.8^‡‡^ | 3.5, 6.5 | 3.8^‡‡‡^ | 2.5, 5.8 | 4.0 | 2.7, 6.1 | 3.9 | 2.9, 5.3 |
| Thinness | 4.4 | 2.7, 7.2 | 6.3^‡‡‡^ | 4.7, 8.3 | 6.7^‡‡‡^ | 4.9, 9.1 | 4.7^‡‡^ | 3.2, 6.9 | 5.7 | 4.5, 7.3 |
| Overweight | 16.5^‡‡‡^ | 12.9, 20.9 | 13.2^‡‡‡^ | 10.9, 15.9 | 13.3^‡‡‡^ | 10.7, 16.4 | 15.2^‡‡‡^ | 12.3, 18.6 | 14.2 | 12.3, 16.5 |
| Obesity | 17.0^‡‡‡^ | 13.3, 21.3 | 15.5^‡‡‡^ | 13.1, 18.3 | 18.3^†,‡‡‡^ | 15.3, 21.8 | 13.6^‡‡‡^ | 10.9, 16.8 | 16.0 | 13.9, 18.3 |
|  |  |  |  |  |  |  |  |  |  |  |
| <5 years (0.5-4.9 years) |  |  |  |  |  |  |  |  |  |  |
| Stunting | 4.9 | 3.4, 7.0 | 6.8 | 5.5, 8.3 | 7.2 | 5.7, 9.1 | 5.1 | 3.8, 6.7 | 6.2 | 5.2, 7.4 |
| Underweight | 4.2 | 2.8, 6.2 | 4.2 | 3.2, 5.5 | 3.2^†^ | 2.2, 4.6 | 5.3 | 4.0, 7.0 | 4.2 | 3.3, 5.2 |
| Wasting | 2.2 | 1.2, 3.7 | 2.1 | 1.4, 3.1 | 2.1 | 1.3, 3.3 | 2.2 | 1.4, 3.4 | 2.1 | 1.5, 2.9 |
| Overweight | 5.5 | 3.9, 7.7 | 5.4 | 4.3, 6.9 | 6.3 | 4.8, 8.1 | 4.6 | 3.4, 6.1 | 5.4 | 4.5, 6.6 |
| Obesity | 2.8 | 1.7, 4.4 | 3.6 | 2.7, 4.8 | 3.7 | 2.6, 5.1 | 3.0 | 2.1, 4.3 | 3.3 | 2.6, 4.3 |
|  |  |  |  |  |  |  |  |  |  |  |
| Overall (0.5-12.9 years) |  |  |  |  |  |  |  |  |  |  |
| Stunting | 3.5^*^ | 2.5, 4.9 | 5.1 | 4.2, 6.2 | 4.8 | 3.9, 6.1 | 4.3 | 3.3, 5.6 | 4.6 | 3.9, 5.4 |
| Underweight (0.5-4.9 y) | 4.2 | 2.8, 6.2 | 4.2 | 3.2, 5.5 | 3.2^†^ | 2.2, 4.6 | 5.3 | 4.0, 7.0 | 4.2 | 3.3, 5.2 |
| Wasting (0.5-4.9 y) | 2.2 | 1.2, 3.7 | 2.1 | 1.4, 3.1 | 2.1 | 1.3, 3.3 | 2.2 | 1.4, 3.4 | 2.1 | 1.5, 2.9 |
| Thinness (5-12.9 y) | 4.5 | 2.9, 6.8 | 5.9 | 4.6, 7.6 | 6.5 | 4.9, 8.6 | 4.4 | 3.1, 6.2 | 5.5 | 4.4, 6.8 |
| Overweight | 12.2 | 10.0, 15.0 | 10.6 | 9.1, 12.3 | 10.8 | 9.1, 12.8 | 11.4 | 9.6, 13.6 | 11.1 | 9.9, 12.5 |
| Obesity | 11.6 | 9.4, 14.3 | 11.8 | 10.3, 13.6 | 13.3^†^ | 11.4, 15.5 | 10.1 | 8.4, 12.1 | 11.8 | 10.5, 13.2 |

^*^*P*<0.05: significant difference between the urban and rural children based on complex samples Pearson Chi-Square.

^†^*P*<0.05, ^††^*P*<0.01: significant difference between the boys and girls based on complex samples Pearson Chi-Square.

^‡^*P*<0.05, ^‡‡^*P*<0.01, ^‡‡‡^*P*<0.001: significance of trend of prevalence of nutritional status with age group in each residence and sex.

Stunting, height-for-age *z*-scores <-2 SD; underweight (under 5 years only), weight-for-age *z*-scores <-2 SD; wasting (under 5 years only), weight-for-height *z*-scores <-2 SD; thinness (5-12 years only), BMI-for-age *z*-scores <-2 SD; overweight, BMI-for-age *z*-scores >2 SD in children aged <5 years and >1 SD in children aged 5-12 years; obesity, BMI-for-age *z*-scores >3 SD in children aged <5 years and >2 SD in children aged 5-12 years.

**Supplementary Table 5** Weighted macronutrient intakes (mean and SE) by age group, residence, and sex

| Variables | EAR | | Residence | | | | Sex | | | | All | |
| --- | --- | --- | --- | --- | --- | --- | --- | --- | --- | --- | --- | --- |
|  | Boys | Girls | Urban | | Rural | | Boys | | Girls | |  |  |
|  |  |  | Mean | SE | Mean | SE | Mean | SE | Mean | SE | Mean | SE |
| 0.5-0.9 years |  |  |  |  |  |  |  |  |  |  |  |  |
| Energy (kJ) |  |  | 3113 | 100 | 3035 | 75 | 3273^†††^ | 93 | 2851 | 72 | 3061 | 60 |
| Energy (kcal) | 680 | 610 | 744 | 24 | 725 | 18 | 782^†††^ | 22 | 681 | 17 | 732 | 14 |
| Carbohydrate (g) |  |  | 96.7 | 3.3 | 94.0 | 2.3 | 100.3^††^ | 2.8 | 89.5 | 2.4 | 94.9 | 1.9 |
| Protein (g) | 10.0 | 8.6 | 22.4 | 1.1 | 21.8 | 0.8 | 24.1^†††^ | 1.0 | 19.9 | 0.8 | 22.0 | 0.7 |
| Fat (g) |  |  | 29.5 | 1.1 | 28.9 | 0.9 | 31.4^†††^ | 1.1 | 26.9 | 0.8 | 29.1 | 0.7 |
| *Percentage of energy* |  |  |  |  |  |  |  |  |  |  |  |  |
| Carbohydrate (%) |  |  | 52.3 | 0.7 | 52.6 | 0.5 | 52.1 | 0.6 | 52.9 | 0.6 | 52.5 | 0.4 |
| Protein (%) |  |  | 11.7 | 0.3 | 11.7 | 0.3 | 12.0 | 0.3 | 11.3 | 0.3 | 11.7 | 0.2 |
| Fat (%) |  |  | 36.0 | 0.6 | 35.7 | 0.5 | 35.8 | 0.6 | 35.7 | 0.5 | 35.8 | 0.4 |
| Dietary fiber (g) |  |  | 2.5 | 0.2 | 2.2 | 0.1 | 2.5^†^ | 0.2 | 2.1 | 0.2 | 2.3 | 0.1 |
|  |  |  |  |  |  |  |  |  |  |  |  |  |
| 1.0-3.9 years |  |  |  |  |  |  |  |  |  |  |  |  |
| Energy (kJ) |  |  | 5072 | 89 | 5076 | 60 | 5181^††^ | 73 | 4954 | 66 | 5075 | 50 |
| Energy (kcal) | 1050 | 980 | 1212 | 21 | 1213 | 14 | 1238^††^ | 17 | 1184 | 16 | 1213 | 12 |
| Carbohydrate (g) |  |  | 147.1 | 2.9 | 149.2 | 2.1 | 152.5^††^ | 2.5 | 144.0 | 2.2 | 148.5 | 1.7 |
| Protein (g) | 11.4 | 10.7 | 48.0 | 1.0 | 47.4 | 0.7 | 48.3 | 0.8 | 46.7 | 0.8 | 47.6 | 0.6 |
| Fat (g) |  |  | 47.6 | 1.1 | 47.1 | 0.7 | 48.0 | 0.8 | 46.4 | 0.8 | 47.2 | 0.6 |
| *Percentage of energy* |  |  |  |  |  |  |  |  |  |  |  |  |
| Carbohydrate (%) |  |  | 49.0 | 0.5 | 49.5 | 0.4 | 49.5 | 0.4 | 49.1 | 0.4 | 49.3 | 0.3 |
| Protein (%) |  |  | 15.8 | 0.2 | 15.7 | 0.1 | 15.6 | 0.1 | 15.8 | 0.1 | 15.7 | 0.1 |
| Fat (%) |  |  | 35.2 | 0.4 | 34.9 | 0.3 | 34.8 | 0.3 | 35.1 | 0.4 | 35.0 | 0.2 |
| Dietary fiber (g) |  |  | 5.1 | 0.2 | 5.2 | 0.1 | 5.3 | 0.2 | 5.0 | 0.1 | 5.2 | 0.1 |
|  |  |  |  |  |  |  |  |  |  |  |  |  |
| 4.0-6.9 years |  |  |  |  |  |  |  |  |  |  |  |  |
| Energy (kJ) |  |  | 6236 | 136 | 6365 | 101 | 6584^†††^ | 122 | 6042 | 104 | 6325 | 82 |
| Energy (kcal) | 1290, 1440^‡^ | 1200, 1320^‡^ | 1490 | 33 | 1521 | 24 | 1574^†††^ | 29 | 1444 | 25 | 1512 | 19 |
| Carbohydrate (g) |  |  | 186.6 | 5.3 | 194.9 | 3.5 | 201.1^††^ | 4.6 | 182.8 | 3.3 | 192.3 | 2.9 |
| Protein (g) | 13.6, 17.1^‡^ | 13.6, 17.1^‡^ | 56.8 | 1.4 | 57.9 | 1.1 | 59.4^†^ | 1.2 | 55.6 | 1.2 | 57.6 | 0.9 |
| Fat (g) |  |  | 56.9 | 1.7 | 56.1 | 1.2 | 58.6^†^ | 1.5 | 54.0 | 1.3 | 56.4 | 1.0 |
| *Percentage of energy* |  |  |  |  |  |  |  |  |  |  |  |  |
| Carbohydrate (%) |  |  | 50.1 | 0.8 | 51.8 | 0.4 | 51.3 | 0.6 | 51.2 | 0.5 | 51.3 | 0.4 |
| Protein (%) |  |  | 15.5 | 0.2 | 15.5 | 0.2 | 15.4 | 0.2 | 15.5 | 0.2 | 15.5 | 0.1 |
| Fat (%) |  |  | 34.4^*^ | 0.6 | 32.8 | 0.4 | 33.3 | 0.5 | 33.3 | 0.4 | 33.3 | 0.3 |
| Dietary fiber (g) |  |  | 6.0 | 0.2 | 6.1 | 0.2 | 6.2 | 0.2 | 6.0 | 0.2 | 6.1 | 0.1 |
|  |  |  |  |  |  |  |  |  |  |  |  |  |
| 7.0-12.9 years |  |  |  |  |  |  |  |  |  |  |  |  |
| Energy (kJ) |  |  | 7234 | 133 | 7305 | 88 | 7636^†††^ | 106 | 6919 | 99 | 7283 | 73 |
| Energy (kcal) | 1440, 1800^‡‡^ | 1320, 1650^‡‡^ | 1729 | 32 | 1746 | 21 | 1825^†††^ | 25 | 1654 | 24 | 1741 | 18 |
| Carbohydrate (g) |  |  | 225.4 | 4.2 | 233.4 | 3.0 | 242.0^†††^ | 3.5 | 219.6 | 3.3 | 231.0 | 2.4 |
| Protein (g) | 17.1, 27.8^‡‡^ | 17.1, 28.6^‡‡^ | 68.1 | 1.7 | 65.7 | 1.0 | 70.1^†††^ | 1.3 | 62.6 | 1.2 | 66.4 | 0.9 |
| Fat (g) |  |  | 60.9 | 1.7 | 60.4 | 1.0 | 63.4^†††^ | 1.3 | 57.7 | 1.2 | 60.6 | 0.9 |
| *Percentage of energy* |  |  |  |  |  |  |  |  |  |  |  |  |
| Carbohydrate (%) |  |  | 53.3 | 0.6 | 54.2 | 0.3 | 53.9 | 0.4 | 53.9 | 0.4 | 53.9 | 0.3 |
| Protein (%) |  |  | 15.7^**^ | 0.2 | 15.1 | 0.1 | 15.3 | 0.2 | 15.2 | 0.2 | 15.3 | 0.1 |
| Fat (%) |  |  | 31.0 | 0.5 | 30.8 | 0.3 | 30.8 | 0.4 | 30.9 | 0.4 | 30.8 | 0.3 |
| Dietary fiber (g) |  |  | 7.4 | 0.2 | 7.0 | 0.1 | 7.2 | 0.2 | 7.1 | 0.2 | 7.1 | 0.1 |

^*^*P*<0.05, ^**^*P*<0.01: significant difference between the urban and rural children based on complex samples ANCOVA after correcting for age.

^†^*P*<0.05, ^††^*P*<0.01, ^†††^*P*<0.001: significant difference between the boys and girls based on complex samples ANCOVA after correcting for age.

^‡^Values are for children aged 4-5 years, children aged 6-8 years.

^‡‡^Values are for children aged 6-8 years, children aged 9-12 years.

Acceptable Macronutrient Distribution Range (AMDR): children aged 0.5-1.9 years, CHO:PRO:FAT 45-65:10-15:35-40; children aged 2.0-12.9 years, CHO:PRO:FAT 45-65:10-15:25-35.

**Supplementary Table 6** Weighted selected micronutrient intakes (mean and SE) by age group, residence, and sex

| Variables | EAR | | Residence | | | | Sex | | | | All | |
| --- | --- | --- | --- | --- | --- | --- | --- | --- | --- | --- | --- | --- |
|  | Boys | Girls | Urban | | Rural | | Boys | | Girls | |  |  |
|  |  |  | Mean | SE | Mean | SE | Mean | SE | Mean | SE | Mean | SE |
| 0.5-0.9 years |  |  |  |  |  |  |  |  |  |  |  |  |
| Calcium (mg) | NA | NA | 427.6 | 27.4 | 451.0 | 22.5 | 527.5^†††^ | 26.9 | 360.2 | 20.6 | 443.3 | 17.6 |
| Iron (mg) | 7.5 | 7.5 | 6.4 | 0.5 | 6.7 | 0.4 | 7.9^†††^ | 0.5 | 5.3 | 0.3 | 6.6 | 0.3 |
| Zinc (mg) | 2.27 | 2.27 | 4.0 | 0.2 | 4.4 | 0.2 | 4.9^†††^ | 0.2 | 3.6 | 0.2 | 4.2 | 0.2 |
| Vitamin A (RAE) | 190.0 | 175.0 | 1241.5 | 175.8 | 978.7 | 77.4 | 1227.2^†^ | 123.4 | 904.1 | 93.6 | 1064.6 | 77.9 |
| Vitamin B12 (µg) | NA | NA | 2.2 | 0.2 | 1.8 | 0.1 | 2.2^†^ | 0.2 | 1.7 | 0.2 | 1.9 | 0.1 |
| Vitamin C (mg) | 49.0 | 49.0 | 69.7 | 4.6 | 70.8 | 3.6 | 79.4^††^ | 4.3 | 61.5 | 3.6 | 70.4 | 2.8 |
| Vitamin D (µg) | NA | NA | 5.0 | 0.5 | 5.3 | 0.4 | 5.9^†^ | 0.4 | 4.5 | 0.3 | 5.2 | 0.3 |
|  |  |  |  |  |  |  |  |  |  |  |  |  |
| 1.0-3.9 years |  |  |  |  |  |  |  |  |  |  |  |  |
| Calcium (mg) | 417^‡^ | 417^‡^ | 641.4 | 19.5 | 681.4 | 14.0 | 672.4 | 16.2 | 664.4 | 15.9 | 668.6 | 11.4 |
| Iron (mg) | 4.3 | 4.3 | 7.5 | 0.3 | 7.2 | 0.2 | 7.3 | 0.3 | 7.2 | 0.2 | 7.3 | 0.2 |
| Zinc (mg) | 3.7 | 3.7 | 4.3 | 0.1 | 4.1 | 0.1 | 4.3 | 0.1 | 4.0 | 0.1 | 4.2 | 0.1 |
| Vitamin A (RAE) | 230.0 | 220.0 | 658.6 | 50.4 | 616.6 | 31.4 | 616.5 | 36.7 | 645.4 | 39.1 | 630.0 | 26.8 |
| Vitamin B12 (µg) | 0.7 | 0.7 | 2.6 | 0.1 | 2.4 | 0.1 | 2.3^†^ | 0.1 | 2.7 | 0.1 | 2.5 | 0.1 |
| Vitamin C (mg) | 20.0 | 20.0 | 40.8 | 2.5 | 40.9 | 1.7 | 39.7 | 1.9 | 42.2 | 2.1 | 40.9 | 1.4 |
| Vitamin D (µg) | 10.0 | 10.0 | 6.1 | 0.2 | 6.1 | 0.1 | 6.1 | 0.2 | 6.1 | 0.2 | 6.1 | 0.1 |
|  |  |  |  |  |  |  |  |  |  |  |  |  |
| 4.0-6.9 years |  |  |  |  |  |  |  |  |  |  |  |  |
| Calcium (mg) | 667^‡^ | 667^‡^ | 553.5 | 21.0 | 538.2 | 18.2 | 548.9 | 17.8 | 536.4 | 22.2 | 542.9 | 14.1 |
| Iron (mg) | 4.9, 5.5^§^ | 4.9, 5.5^§^ | 6.9 | 0.2 | 6.9 | 0.2 | 7.0 | 0.2 | 6.9 | 0.2 | 6.9 | 0.1 |
| Zinc (mg) | 4.46, 5.22^§^ | 4.46, 5.22^§^ | 4.0^*^ | 0.1 | 4.4 | 0.1 | 4.5^†^ | 0.1 | 4.1 | 0.1 | 4.3 | 0.1 |
| Vitamin A (RAE) | 335.0, 350.0^§^ | 340.0 | 455.4 | 34.1 | 416.9 | 22.4 | 420.4 | 25.2 | 437.9 | 27.7 | 428.8 | 18.7 |
| Vitamin B12 (µg) | 1.0 | 1.0 | 2.4 | 0.1 | 2.4 | 0.1 | 2.4 | 0.1 | 2.4 | 0.1 | 2.4 | 0.1 |
| Vitamin C (mg) | 25.0, 30.0^§^ | 25.0, 30.0^§^ | 32.8 | 3.2 | 29.0 | 1.6 | 31.3 | 2.4 | 29.0 | 1.6 | 30.2 | 1.5 |
| Vitamin D (µg) | 10.0 | 10.0 | 5.0 | 0.2 | 5.1 | 0.2 | 5.1 | 0.2 | 5.1 | 0.2 | 5.1 | 0.1 |
|  |  |  |  |  |  |  |  |  |  |  |  |  |
| 7.0-12.9 years |  |  |  |  |  |  |  |  |  |  |  |  |
| Calcium (mg) | 667, 833^‡,\|\|^ | 667, 833^‡,\|\|^ | 399.2 | 15.9 | 411.8 | 8.9 | 415.9 | 10.5 | 399.7 | 11.7 | 407.9 | 7.9 |
| Iron (mg) | 5.5, 9.9^\|\|^ | 5.5, 10.4, 13.0^¶^ | 7.8 | 0.2 | 8.0 | 0.2 | 8.2^†^ | 0.2 | 7.6 | 0.2 | 7.9 | 0.1 |
| Zinc (mg) | 5.22, 7.94^\|\|^ | 5.22, 7.53^\|\|^ | 5.0 | 0.1 | 5.1 | 0.1 | 5.4^†††^ | 0.1 | 4.7 | 0.1 | 5.0 | 0.1 |
| Vitamin A (RAE) | 350.0, 395.0^\|\|^ | 340.0, 405.0^\|\|^ | 419.9 | 74.9 | 494.2 | 39.9 | 526.9 | 61.6 | 413.7 | 36.1 | 471.2 | 36.1 |
| Vitamin B12 (µg) | 1.0, 1.5^\|\|^ | 1.0, 1.5^\|\|^ | 2.4 | 0.1 | 2.5 | 0.1 | 2.6 | 0.1 | 2.4 | 0.1 | 2.5 | 0.1 |
| Vitamin C (mg) | 30.0, 50.0^\|\|^ | 30.0, 50.0^\|\|^ | 35.9 | 2.9 | 30.9 | 1.2 | 31.1 | 1.8 | 33.7 | 1.6 | 32.4 | 1.2 |
| Vitamin D (µg) | 10.0 | 10.0 | 3.9 | 0.2 | 4.1 | 0.1 | 4.1 | 0.1 | 4.0 | 0.1 | 4.1 | 0.1 |

^*^*P*<0.05: significant difference between the urban and rural children based on complex samples ANCOVA after correcting for age.

^†^*P*<0.05, ^††^*P*<0.01, ^†††^*P*<0.001: significant difference between the boys and girls based on complex samples ANCOVA after correcting for age.

^‡^Harmonized Average Requirement (H-AR)

^§^Values are for children aged 4-5 years, children aged 6-8 years.

^||^Values are for children aged 6-8 years, children aged 9-12 years.

^¶^Values are for children aged 6-8 years, boys aged 9-12 years, girls aged 9-12 years.
